# Genome-wide identification of WRKY transcription factors in *Casuarina equisetifolia* and the function analysis of CeqWRKY11 in response to NaCl/NaHCO_3_ stresses

**DOI:** 10.1186/s12870-024-04889-w

**Published:** 2024-05-08

**Authors:** Xiaohong Zhao, Guoning Qi, Jinhong Liu, Kui Chen, Xinxin Miao, Jamshaid Hussain, Shenkui Liu, Huimin Ren

**Affiliations:** 1 State Key Laboratory of Subtropical Silviculture, Zhejiang A & F University, Hangzhou, Zhejiang 311300 China; 2 Department of Biotechnology, COMSATS University Islamabad, Abbottabad Campus, University Road, Tobe Camp, Abbottabad, 22060 Pakistan

**Keywords:** WRKY transcription factor, Expression pattern, Function analysis, NaCl, NaHCO_3_ stress, *Casuarina equisetifolia*

## Abstract

**Background:**

*Casuarina equisetifolia* (*C. equisetifolia*) is a woody species with many excellent features. It has natural resistance against drought, salt and saline-alkali stresses. WRKY transcription factors (TFs) play significant roles in plant response to abiotic stresses, therefore, molecular characterization of *WRKY* gene family under abiotic stresses holds great significance for improvement of forest trees through molecular biological tools. At present, WRKY TFs from *C. equisetifolia* have not been thoroughly studied with respect to their role in salt and saline-alkali stresses response. The current study was conducted to bridge the same knowledge gap.

**Results:**

A total of 64 *WRKYs* were identified in *C. equisetifolia* and divided into three major groups i.e. group I, II and III, consisting of 10, 42 and 12 *WRKY* members, respectively. The *WRKY* members in group II were further divided into 5 subgroups according to their homology with *Arabidopsis* counterparts. *WRKYs* belonging to the same group exhibited higher similarities in gene structure and the presence of conserved motifs. Promoter analysis data showed the presence of various response elements, especially those related to hormone signaling and abiotic stresses, such as ABRE (ABA), TGACG (MeJA), W-box ((C/T) TGAC (T/C)) and TC-rich motif. Tissue specific expression data showed that *CeqWRKYs* were mainly expressed in root under normal growth conditions. Furthermore, most of the *CeqWRKYs* were up-regulated by NaCl and NaHCO_3_ stresses with few of *WRKYs* showing early responsiveness to both stresses while few others exhibiting late response. Although the expressions of *CeqWRKYs* were also induced by cold stress, the response was delayed compared with other stresses. Transgenic *C. equisetifolia* plants overexpressing *CeqWRKY11* displayed lower electrolyte leakage, higher chlorophyll content, and enhanced tolerance to both stresses. The higher expression of abiotic stress related genes, especially *CeqHKT1* and *CeqPOD7*, in overexpression lines points to the maintenance of optimum Na^+^/K^+^ ratio, and ROS scavenging as possible key molecular mechanisms underlying salt stress tolerance.

**Conclusions:**

Our results show that *CeqWRKYs* might be key regulators of NaCl and NaHCO_3_ stresses response in *C. equisetifolia*. In addition, positive correlation of *CeqWRKY11* expression with increased stress tolerance in *C. equisetifolia* encourages further research on other WRKY family members through functional genomic tools. The best candidates could be incorporated in other woody plant species for improving stress tolerance.

**Supplementary Information:**

The online version contains supplementary material available at 10.1186/s12870-024-04889-w.

## Introduction

WRKY is one of the biggest transcription factor (TF) family in plants like *Arabidopsis thaliana* [1], *Oryza sativa* [2], *Tritipyrum* [3], and *Isatis indigotica* [4]. WRKY proteins have highly conserved structure, containing one or two WRKY domains composed of approximately 60 amino acid residues. The N-terminal of the domain contains the WRKYGQK sequence, which is related to DNA binding activity, while the C-terminal of the domain includes the C-X4-5-C-X22-23-H-X1-H or C-X7-C-X23-H-X1-C zinc finger, which participates in protein interaction and improves DNA binding [4, 5].

WRKY transcription factors play important roles in regulating plant growth, development, and response to drought, temperature and salt stresses [6, 7]. For example, *OsWRKY45* plays a positive role, whereas both *OsWRKY5* and *OsWRKY114* separately play negative roles in response to drought stress [8–10]. Overexpression of *BdWRKY36* and *PheWRKY86* improved drought tolerance in tobacco and rice, respectively [11, 12]. Additionally, it has been demonstrated that *KoWRKY40* and *LlWRKY22* participate in response to temperature stress [13, 14]. At present, a large number of salt responsive WRKY transcription factors have been identified in various plants. Overexpression of *FcWRKY40* in tobacco, and overexpression of *VvWRKY30* and *GmWRKY49* in *A. thaliana* confer salt tolerance in transgenic plants [15–17]. *ZmWRKY86* and *OsWRKY28*, *OsWRKY54* also play important roles in improving maize and rice salt tolerance, respectively [18–20]. In addition, *PtWRKY39* and *PsnWRKY70* play significant positive and negative roles in saline-alkali stress response, respectively [21, 22]. Moreover, it has been demonstrated that the interactions of different WRKYs, as well as WRKY and other TFs also play indispensable roles in modulating plant salt stress response [23, 24]. The latest research showed that ZmWRKY20 interacts with ZmWRKY115 to repress the expression of *ZmbZIP111*, thus increasing the salt sensitivity of maize seedlings [25].

*C. equisetifolia* is a typical tropical and subtropical woody plant and exhibits drought and salt/saline-alkali resistance. It has reported that *C. equisetifolia* seedlings can survive in 500 mM NaCl solution [26]. In addition, *C. equisetifolia* plays significant roles in wind prevention, sand fixation, and soil improvement [27, 28]. With the publication of the *C. equisetifolia* genome data and establishment of the regeneration and genetic transformation systems, it is possible to study the molecular mechanisms of stress tolerance in *C. equisetifolia* [29, 30]. In recent years, there has been increasing research on the mechanism of *C. equisetifolia* response to stress, especially in response to salt stress. For example, *CeqSnRK*, *CeqMYB* and *CeqHAK* gene families are involved in response to salt stress [31–33]. However, little research has been conducted regarding the role of *WRKYs* in response to NaCl/NaHCO_3_ stress in *C. equisetifolia*. Therefore, it is essential and significant to identify the salt stress-responsive WRKY TFs in *C. equisetifolia*.

In this study, a total of 64 *WRKY* genes were identified in *C. equisetifolia* using the known *AtWRKY* gene sequences as query. Then, physicochemical properties, phylogenetic relationships, conserved motifs, gene structure, and *cis*-acting elements were performed. Further, the expression levels of all *CeqWRKY* genes in roots and shoots under different abiotic stresses (cold, NaCl, NaHCO_3_, and pH) at different treatment time points were detected by using transcriptome data and qRT-PCR, respectively. In addition, based on the transcriptome data, the co-expression networks were also analyzed and several key *CeqWRKY* genes were identified, including *CeqWRKY11*, *CeqWRKY33*, *CeqWRKY41* and *CeqWRKY46*. We further investigated the subcellular localization and transactivation activity of these CeqWRKYs. Finally, we got the transgenic plants with overexpression of *CeqWRKY11* and further analyzed its physiological function in response to NaCl and NaHCO_3_ stresses by phenotype detection and the expression analysis of several salt-responsive genes (*CeqHKT1*, *CeqNHX, CeqRD29A*, *CeqUGTs*) as well as oxidative related genes (*CeqPOD*, *CeqRbohD*, *CeqCSD1*, *CeqGols*) in the *C. equisetifolia* transgenic plants. Consequently, our study not only provided a new perspective to explore the functions of *WRKY* genes in *C. equisetifolia* to response abiotic stresses, but also helped to choose candidate genes for molecular improvement of forest trees.

## Materials and methods

### Plant materials, growth conditions, and treatments

The *C. equisetifolia* seedlings were selected from the plant tissue culture room at 25 ℃ with 16 h/8 h light/dark cycle in Zhejiang Agriculture and Forestry University. The stem segments were cut from the selected seedlings and grew in the rooting medium for about two months [30]. The regenerated seedlings with similar size (about 15 cm in height) were transferred into hydroponic solution for different treatments, including 1/2 Hoagland nutrient solution (CK), 1/2 Hoagland nutrient solution with 300 mM NaCl, 300 mM NaHCO_3_, and pH = 8.5, respectively. Roots and shoots were harvested for RNA-seq experiments at 0, 0.5, 5, 12 and 24 h time points under each treatment and each time point included three biological replicates. Then, these samples were immediately frozen in liquid nitrogen, and transferred to an ultra-low temperature freezer for storage at -80 °C for RNA extraction and sequencing.

For phenotype analysis, the seedlings of *CeqWRKY11* overexpression lines (*CeqWRKY11OE-1* and *CeqWRKY11OE-2*) generated by using hairy root transgenic system [30] and control plants (transformed with empty vector, named as CK) were transplanted in 1/2 Hoagland nutrient solution, and cultivated in plant greenhouse at 25 ℃ with 16 h/8 h light/dark cycle for about two months. During the process, the nutrient solution were replaced each week. Then, these plants with about 15 cm in height were treated with 200 mM NaCl for 5 days or 75 mM NaHCO_3_ for 15 days. The electronic leakage and chlorophyll content were analyzed using the methods described by Dahro et al. [34, 35]. Three biological replicates were performed.

For subcellular localization analysis, the seeds of tobacco were sowed in the pot and grew in greenhouses at 25 ℃ with 16 h/8 h light/dark cycle for one month. Then, the leaves were used for subcellular localization experiment.

### Sequence retrieval and gene identification

The *C. equisetifolia* genome data was downloaded from the website (http://forestry.fafu.edu.cn/db/Casuarinaceae/). WRKY sequences of *A. thaliana* were downloaded from The Arabidopsis Information Resource (TAIR) database (https://arabidopsis.org/browse/genefamily/WRKY.jsp). Pfam protein family database (http://pfam.xfam.org/) was used to download the hidden MarKov model (HMM) file related WRKY domain (PF03106), which was used as query (*P* < 0.001) to search all putative CeqWRKY protein [32]. Then, all candidate *WRKY*s screened by the Pfam database (http://pfam.janelia.org/), were further identified in NCBI conserved domain database (http://www.ncbi.nlm.nih.gov/Structure/cdd/wrpsb.cgi) and SMART database (https://smart.embl.de/). The number of amino acids, molecular weight (MW) and isoelectric point (pI) of each WRKY protein were analyzed using ExPASy (https://www.expasy.org/).

### Phylogenetic tree construction

Putative WRKY proteins in *C. equisetifolia* were aligned with AtWRKYs using ClustalX 2.11 software with default parameters [36, 37]*.* According to the alignment, a neighbor-joining (NJ) phylogenetic analysis was performed by MEGA7. Bootstrap analysis with 1000 replicates was conducted to calculate the reliability of the NJ tree [38].

### Structural and conserved motif analysis of CeqWRKYs

To determine the exon–intron organization, the full-length genomic sequence of candidate *CeqWRKY* genes was analyzed by using TBtools. The MEME online program (http://meme-suite.org/tools/meme) was applied to analyze the conserved motifs in WRKY proteins in *C. equisetifolia*. The parameters used in MEME online program were as followed: maximum number of motifs, 20; and optimum motif length, 6–200 residues.

### Expression profiling of *CeqWRKY* genes

Transcriptome data (Reads Per Kilobase per Million mapped reads; RPKM) were analyzed to investigate the expression profiles of *CeqWRKY* genes in roots and shoots under different treatments (300 mM NaCl, 300 mM NaHCO_3_ and pH 8.5), at different time points. Heatmaps was generated using the TBtools software [39].

### RNA extraction and qRT-PCR analysis

Total RNA was extracted from plant roots and shoots using the RNAprep Pure Plant Plus Kit (Tiangen Biotech, Beijing, China). The integrity and concentration of the RNA was determined by NanoDrop™ One/OneC (ThermoFisher Scientific, USA). The first strand cDNA was synthesized by HiScriptII Q RT superMix for qPCR (gDNA wiper) (Vazyme, Nanjing, China) according to the manufacturer’s instructions. qRT-PCR was performed on an CFX 384 TM Real-time PCR Detection System using ChamQTM SYBR Color qPCR Master Mix (Vazyme, Nanjing, China) with a 10 μL sample volume. Each reaction mixture contained 1.0 μL of diluted cDNA, 0.5 μL of each primer, 5.0 μL of qPCR Master Mix, and 3.0 μL of RNase-free water. qPCR reaction cycling conditions were set according to the manufacturer’s instruction for ChamQTM SYBR Color qPCR Master Mix. The relative expression level of each gene was calculated using 2^−ΔΔCT^ method [40] and the expression in non-treated plants was set as 1. The images were generated by using GraphPad Prism8 software [41]. The primers for *CeqWRKY* genes were designed by Primer 3 Input (version 0.4.0) software and the *CeqEF1α* was used as housekeeping gene [32].

### Putative promoter *cis*-acting element analysis and co-expression networks structure

In order to study the regulatory mechanism of the *CeqWRKYs* in response to abiotic stresses, the 2, 000 bp upstream region from the translation start site of the *CeqWRKY* genes was obtained from the *C. equisetifolia* database and *cis*-acting elements were identified by using the PlantCARE (https://bioinformatics.psb.ugent.be/webtools/plantcare/html/) and PlantPAN 3.0 program (PlantPAN 3.0 (ncku.edu.tw)) [42, 43]. Subsequently, we chose 11 representative *cis*-acting elements associated with plant growth and development, hormone response, and environmental stress responses for further analysis.

Through SPSS software, One-way analysis of variance (ANOVA) was used to analyze the Pearson correlation coefficients of the *WRKY*s expression data, and to further construct the co-expression networks. These networks were visualized by Cytoscape (v3.7.2) [44].

### Subcellular localization and transactivation activity assay

The subcellular localization of putative CeqWRKY proteins was predicted using online website WoLP PSORT (https://wolfpsort.hgc.jp/). The coding sequences of *CeqWRKYs* (without the stop codon) were PCR amplified using the primers (Table S6) and then incorporated into the pCAMBIA1300-35S-GFP vector upstream of green fluorescent protein (GFP) gene. The recombinant plasmids (pCAMBIA1300-35S-CeqWRKYs::GFP) were separately transformed to the *Agrobacterium tumefaciens* strain GV3101. The suspension containing recombinant plasmid was injected into the lower epidermis of tobacco leaves and labeled. After two days culture in dark, GFP fluorescence was observed by confocal microscope (LSM710, Carl Zeiss, Jena, Germany).

For transactivation activity analysis, the open reading frames (ORFs) of *CeqWRKYs* were amplified using the primers given in Table S6 and were inserted into the pGBKT7 vector (Clontech) containing the GAL4 DNA-binding domain (BD), to get fusion vectors designated as pGBKT7-CeqWRKYs. The pGAL4 and the pGBKT7 empty vector were used as a positive and negative control, respectively. The control and fusion plasmids were separately transformed into the yeast strain AH109 according to the method of Sun et al. [45]. The transformed cells were streaked on SD/-Trp and SD/-Trp/-His media. The transactivation activity of the CeqWRKY proteins was assessed based on the growth of the yeast cells at 30℃ for 3–5 days. Three biological replicates were performed.

### Generation of transgenic *C. equisetifolia* with overexpressing *CeqWRKY11* by using efficient hairy root transgenic system

The full-length coding sequence (CDS) of *CeqWRKY11* was cloned into the pCAMBIA1300-GFP vector containing the CaMV 35S promoter fused with the green fluorescent protein (GFP) reporter gene and transferred into one month old *C. equisetifolia* seedlings by using hairy root transgenic system [30]. The transgenic positive seedlings were identified through GFP detection by using LUYOR-3415RG (Hand-held Lamp) and the expression analysis of *CeqWRKY11* by qRT-PCR. The primers used were in Table S5.

### Statistical analysis

The significant difference between mean values was determined using T-test by GraphPad Prism8. Different number of asterisks against the error bars of histograms represents significant differences relative to controls, which were indicated at **P < 0.01 and *P < 0.05.

## Results

### Identification of *WRKY* genes in *C. equisetifolia*

The candidate genes with typical WRKY or WRKY-like domains were preliminarily screened from *C. equisetifolia* genomic database according to the Hidden Markov Model (HMM) profile of the WRKY domain. After removing the incomplete domain sequences, a total of 64 *WRKY* genes were identified in *C. equisetifolia*, and were named according to evolutionary relationship with those of *A. thaliana* genes. As shown in Table S1, the lengths of CeqWRKY protein sequences ranged from 131 to 788 amino acids, and molecular weight ranged from 14.56 KDa to 84.76 KDa. Moreover, the theoretical isoelectric point (pI) varied from 4.77 to 9.80 (Table S1). According to the predicted subcellular localization data, most of the CeqWRKY proteins were localized in the nucleus, whereas two (CeqWRKY10 and CeqWRKY70) were localized in both nucleus and cytoplasm (Table S1).

### Phylogenetic trees and group classification of CeqWRKY proteins

**Fig. 1:**
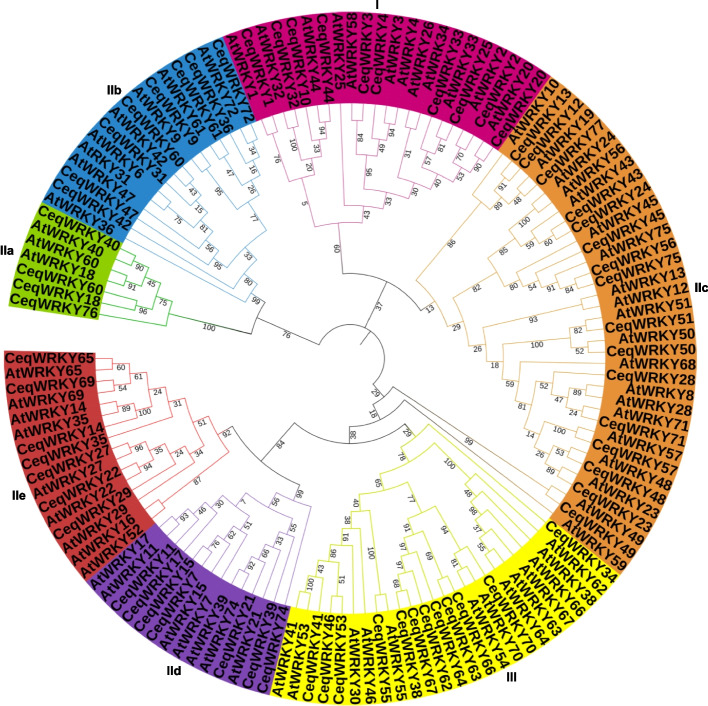
Phylogenetic tree showing evolutionary among WRKY domains of *C. equisetifolia* and *A. thaliana*. The phylogenetic tree was constructed using the neighbor-joining method in the MEGA7 software

According to the alignment, the phylogenetic tree was constructed using CeqWRKYs and AtWRKYs through the MEGA7 software (Fig. 1). As shown in Fig. 1, 64 CeqWRKYs were clustered into three major groups, including group I (10 CeqWRKYs), group II (42 CeqWRKYs), and group III (12 CeqWRKYs). In Group II, CeqWRKY members were further divided into 5 subgroups, including group IIa (4 CeqWRKYs), group IIb (8 CeqWRKYs), group IIc (16 CeqWRKYs), group IId (7 CeqWRKYs) and group IIe (7 CeqWRKYs). The number of CeqWRKY distributed in different groups in *C. equisetifolia* was in accordance with those in *A. thaliana*. The largest subgroup was group IIc, while the smallest was IIa.

### Protein motifs and gene structure analysis

**Fig. 2:**
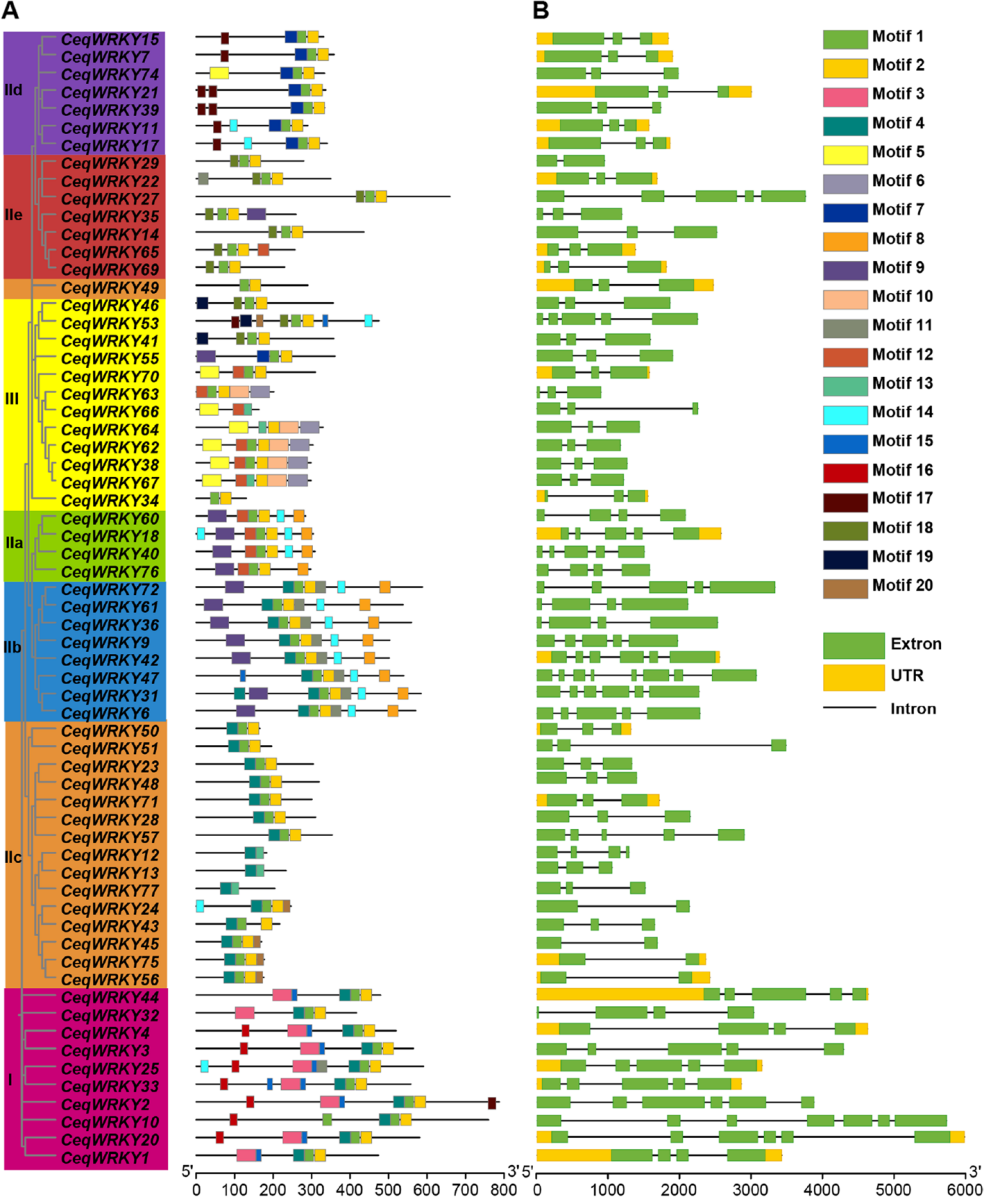
Phylogenetic relationships, conserved motifs and gene structure analysis of CeqWRKYs. **A** Phylogenetic tree and conserved motifs of CeqWRKYs. Different colored boxes represent the presence of 1–20 conserved motifs. **B** Gene structure analysis of *CeqWRKYs*. Introns are depicted by black lines, exons by green boxes, and untranslated 5’- and 3’-regions by yellow boxes

To further analyze the conserved domains of the CeqWRKYs, the MEME online program (http://meme-suite.org/tools/meme) was performed. Overall, 20 conserved motifs were identified in CeqWRKYs; the sequences of these motifs were shown in Table S2. Moreover, we found that CeqWRKY from the same group or subgroup contained similar conserved motifs. In detail, Motif 1 and Motif 3 consisted of the conserved heptapeptide domains (WRKYGQK) of WRKY proteins (Fig. 2A, Table S2). Motif 1 and Motif 2 were distributed in most of the WRKYs, while Motif 3 was only distributed in group I. Motif 8 was unique in group IIa and IIb (Fig. 2A). Although the functions of the conserved motifs have not yet been understood, the classification based on the conserved motifs of the CeqWRKY proteins has widely been used.

As the evolution of multi-gene families is attributed to the diversity of the gene structure, so the exon–intron structure of the 64 *CeqWRKY* genes was analyzed. The exons in genes from group I ranged from 4–7 while those in group IIa, group IIb, and group IIc ranged from 4–5, 4–8, and 2–5, respectively (Fig. 2B). All the members of group IId and most of group IIe and group III contained three exons (Fig. 2B). Genes in the same group generally had similar structures, but significant differences existed in the number of introns and exons in the different groups, indicating that the structure of *CeqWRKY* genes was relatively complex.

### The analysis of *cis*-acting elements in *CeqWRKY* gene promoters

**Fig. 3:**
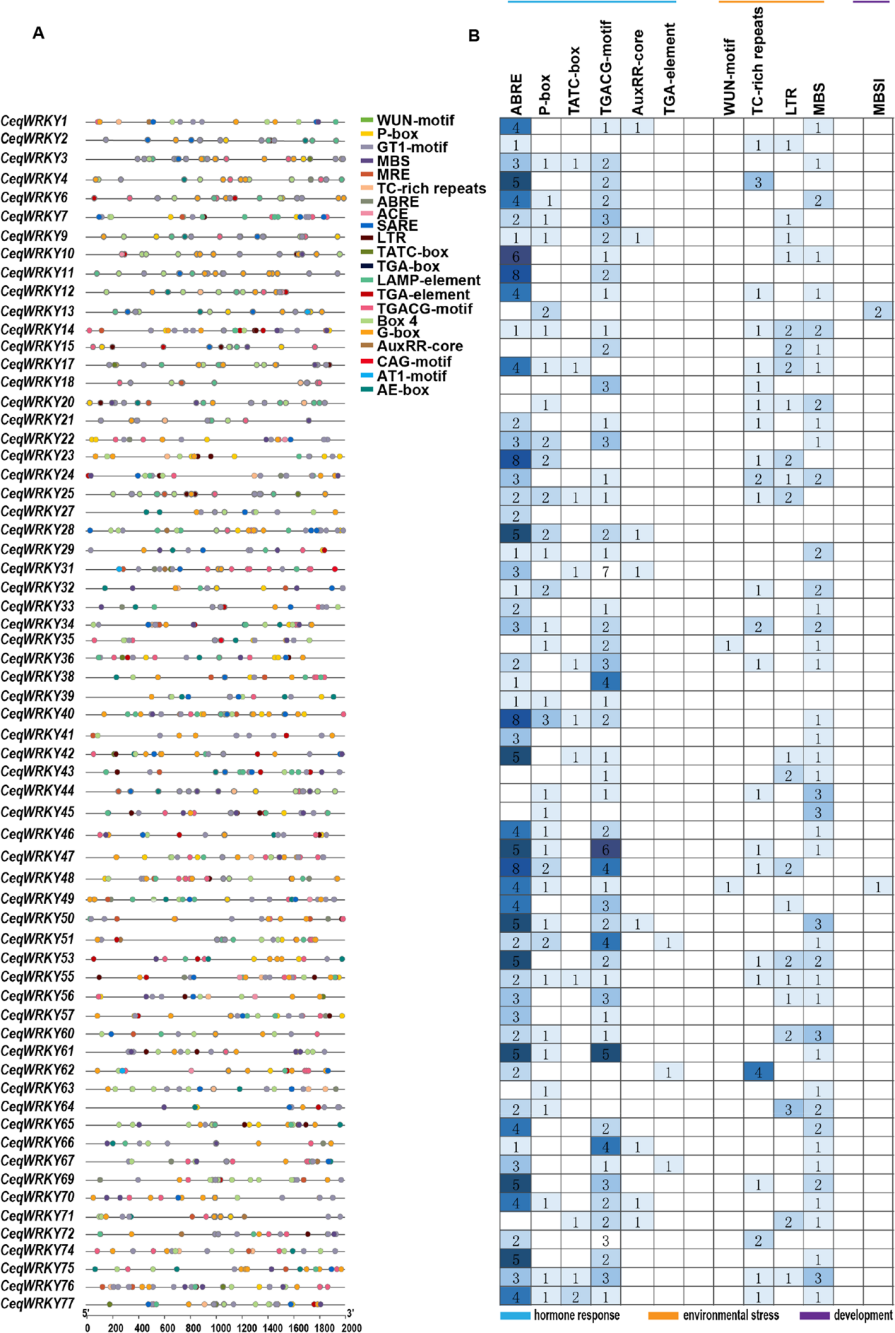
*Cis*-elements analysis in the *CeqWRKYs* promoter region. **A** The number of different types of *cis*-elements in the *CeqWRKYs* promoter region. **B** The number of 11 representative *cis*-acting regulatory elements in promoter regions of *CeqWRKYs*

In order to study the potential regulatory role of CeqWRKY transcription factors in stress responses, the *cis-*acting regulatory elements contained in the promoters of 64 *CeqWRKY* genes were analyzed. The results showed that all *CeqWRKY* genes contained diverse *cis-*acting elements in their promoter regions. Further, the distribution of 11 representative *cis*-acting regulatory elements in promoter regions was investigated. Based on their functions, these *cis*-acting elements were divided into three categories: category 1 included motifs related to hormone response (ABRE, P-box, TATC-box, TGACG-motif, AuxRR-core, TGA-element), and category 2 had motifs related to environmental stress (WUN-motif, TC-rich repeats, LTR and MBS), while category 3 included motifs related to development (MBSI) [32, 46, 47]. The analysis showed that abundant *cis*-acting elements were distributed in the promoter regions of *CeqWRKYs* (Fig. 3A). More than half of *CeqWRKYs* contained 3–5 ABRE elements (Fig. 3B), which play important roles in ABA-mediated diverse stress response in plants [47]. In addition, 52 *CeqWRKYs* contained TGACG-motifs that could be related to MeJA response pathway. Additionally, 34 *CeqWRKYs* containing P-box and 11 *CeqWRKYs* containing TATC-box may be involved in gibberellin response, while 8 *CeqWRKYs* containing AuxRR-core and 3 *CeqWRKYs* containing TGA-element were related to auxin response (Fig. 3B). The promoters of *CeqWRKY* genes also contained a substantial number of environmental stress-related response elements, with 24 *CeqWRKYs* containing TC-rich repeats (Fig. 3B) and all *CeqWRKYs* containing W-box (Fig. S1). Only *CeqWRKY35* and *CeqWRKY49* included WUN-motif (wound-responsive element), which may play critical roles in plant response to mechanical damage. Additionally, many *CeqWRKYs’* promoters included LTR (low-temperature stress responsive element) and MBS (drought stress responsive element) which significantly contribute to plant responses to low temperature and drought stress.

### Determination of expression pattern analysis of *CeqWRKYs* under cold, NaCl, NaHCO_3_ and pH stresses

**Fig. 4:**
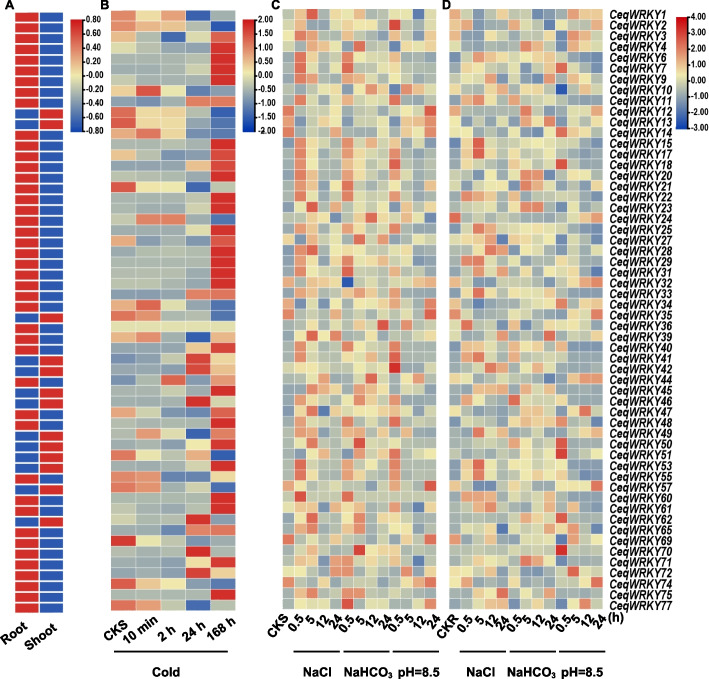
Heatmap of *CeqWRKYs* expression using RNA-seq data. Blue color represents low expression levels, while red color represents high expression levels. **A** The expression patterns of *CeqWRKYs* in root and shoot. **B** The expression of *CeqWRKYs* in shoot under cold stress. **C**-**D** The expression patterns of *CeqWRKYs* in shoot and root under NaCl, NaHCO_3_, and pH stresses

To further investigate the changes in *CeqWRKYs* gene expression under different abiotic stresses, the transcriptome data of *C. equisetifolia* under four different treatments i.e. 300 mM NaCl, 300 mM NaHCO_3_, pH 8.5 and low temperature (4℃), at different time points were analyzed (Fig. 4, Tables S3 and S4). 43 *CeqWRKYs* were highly expressed in root, while 13 *CeqWRKYs* showed strong expression in shoot (Fig. 4A). Under cold treatment, the majority of *CeqWRKYs* displayed increased expression as treatment time increased (Fig. 4B). *CeqWRKY10* and *CeqWRKY44* were strongly up-regulated at 10 min and 2 h time points under low temperature stress. 28 *CeqWRKYs* were significantly induced by cold treatment at 168 h time point (Fig. 4B)*.* According to the expression patterns of *WRKY* genes under NaCl, NaHCO_3_, and pH stresses, we found that some *CeqWRKYs* responded to two stresses. For example, in shoot, *CeqWRKY6*, *CeqWRKY17*, *CeqWRKY22*, *CeqWRKY31*, *CeqWRKY46*, *CeqWRKY50*, and *CeqWRKY60* were induced by both NaCl and NaHCO_3_ stresses, while *CeqWRKY36*, *CeqWRKY40*, *CeqWRKY48*, *CeqWRKY51*, and *CeqWRKY72* were up-regulated by both NaHCO_3_ and pH stresses (Fig. 4C). In root, plenty of *CeqWRKYs* also displayed much higher expression than control under different stresses, such as *CeqWRKY11*, *CeqWRKY33*, and *CeqWRKY41* under NaCl stress, *CeqWRKY39*, *CeqWRKY46*, and *CeqWRKY71* under NaHCO_3_ stress, and *CeqWRKY14*, *CeqWRKY50*, and *CeqWRKY70* under pH stress (Fig. 4D). These results indicated that *WRKYs* could be involved in diverse abiotic stresses response in *C. equisetifolia*.

### Identification of *CeqWRKYs* expression in root and shoot under NaCl and NaHCO_3_ stresses

Previous study has reported that *C. equisetifolia* has strong salt and saline-alkali tolerance [48]. To verify the transcriptional changes and identify the candidate *CeqWRKYs* involved in response to NaCl and NaHCO_3_ conditions, RNA was extracted from the shoot and root of *C. equisetifolia* grown under control, NaCl and NaHCO_3_ treatment at different time points, respectively, and the quality of total RNA was detected (Table S7). Then, the expression of 60 *CeqWRKYs* in root and shoot was determined by qRT-PCR at different time points.

**Fig. 5:**
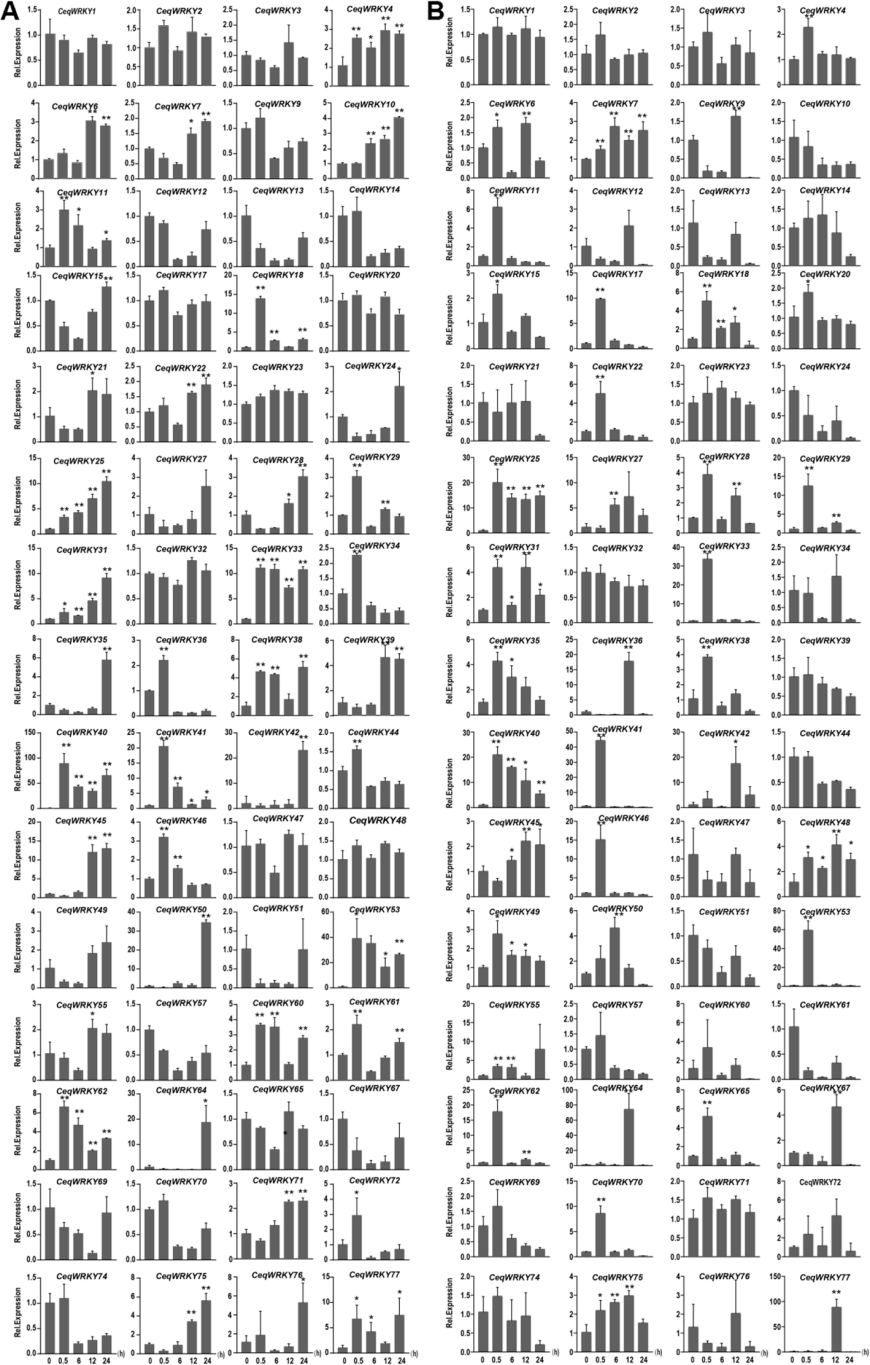
Expression profiles of *CeqWRKYs* under NaCl stress. **A** The expression patterns of *CeqWRKYs* under NaCl stress in root. **B** The expression patterns of *CeqWRKYs* under NaCl stress in shoot. The mean ± standard error measurement (SEM) value with three replications is displayed. Relative expression in untreated plants (0 h) was set to 1. T-tests were used to indicate significant difference: * indicates *p* < 0.05, and ** indicates *p* < 0.01

Under salt treatment, some *CeqWRKYs* such as *WRKY18*, *WRKY29*, *WRKY33*, *WRKY40*, *WRKY41*, displayed significant up-regulation at 0.5 h after the start of the salt treatment, while some *CeqWRKYs* such as *WRKY35*, *WRKY42*, *WRKY50*, and *WRKY64* were induced at 24 h in root (Fig. 5A). *CeqWRKY4*, *CeqWRKY10*, *CeqWRKY33*, *CeqWRKY38*, *CeqWRKY40*, *CeqWRKY53*, and *CeqWRKY62* displayed rapid and sustained response to salt stress in root (Fig. 5A). In shoot, some salt-responsive *CeqWRKYs*, including *CeqWRKY11*, *CeqWRKY17*, *CeqWRKY22*, *CeqWRKY25*, *CeqWRKY33*, *CeqWRKY40*, *CeqWRKY41*, *CeqWRKY46*, *CeqWRKY53*, *CeqWRKY62*, *CeqWRKY70*, and *CeqWRKY77*, showed different degrees of induced expression (Fig. 5B). In addition, *CeqWRKY25* and *CeqWRKY40* showed a persistent high expression at different time points under salt stress in both root and shoot (Fig. 5).

**Fig. 6:**
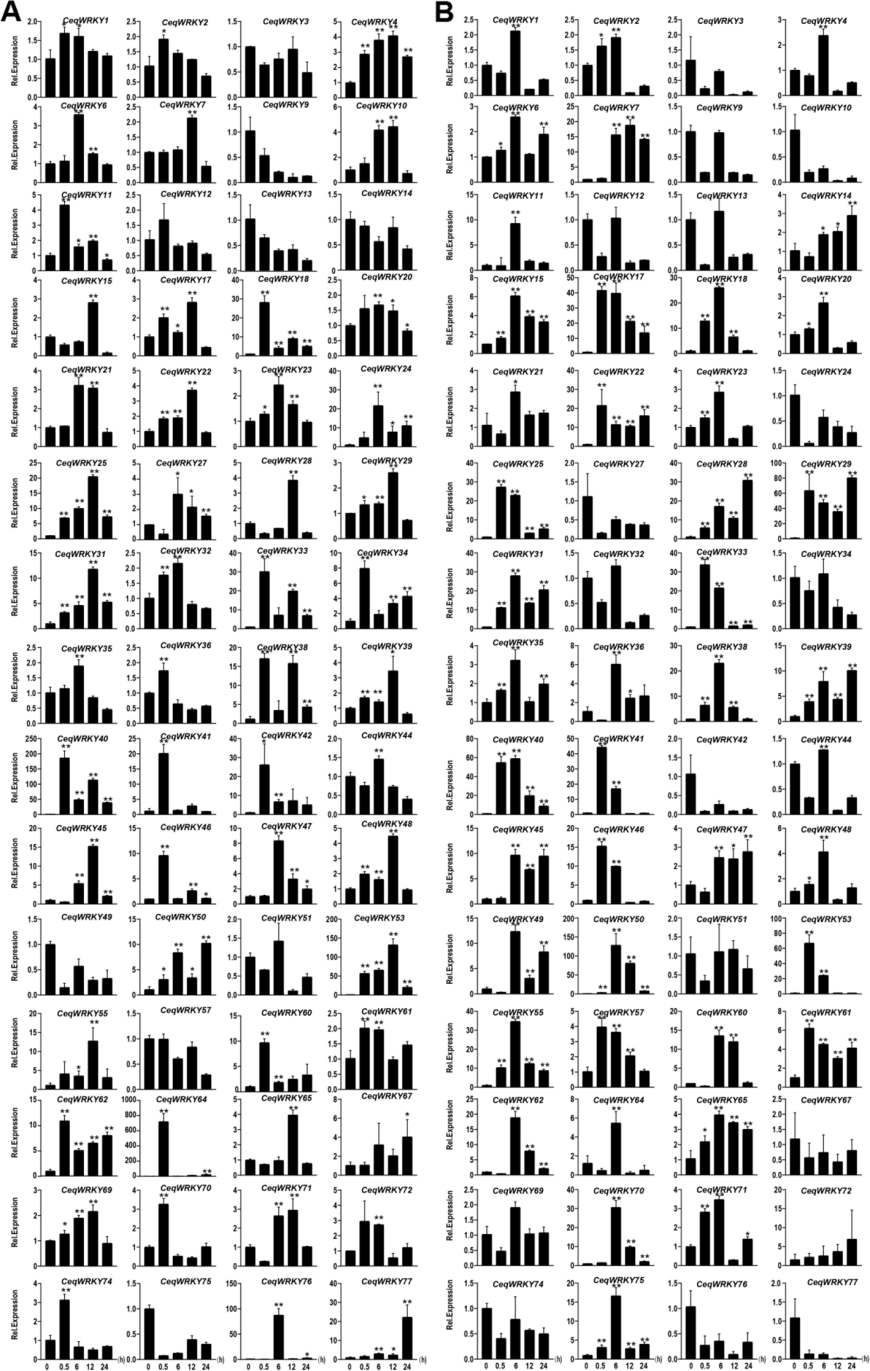
Expression profiles of *CeqWRKYs* under NaHCO_3_ stress. **A** The expression patterns of *CeqWRKYs* under NaHCO_3_ stress in root. **B** The expression patterns of *CeqWRKYs* under NaHCO_3_ stress in shoot. The mean ± standard error measurement (SEM) value with three replications is displayed. Relative expression in untreated plants (0 h) was set to 1. T-tests were used to indicate significant difference: * indicates *p* < 0.05, and ** indicates *p* < 0.01

Under NaHCO_3_ stress, many *CeqWRKYs* showed significant early response (0.5 h) and late response (12 h or 24 h). In roots, *CeqWRKY11*, *CeqWRKY18*, *CeqWRKY33*, *CeqWRKY34*, *CeqWRKY41*, *CeqWRKY46*, *CeqWRKY64*, and *CeqWRKY70* were obviously up-regulated at early treatment time point, while *CeqWRKY15*, *CeqWRKY28*, *CeqWRKY48*, *CeqWRKY65* and *CeqWRKY77* responded to the NaHCO_3_ stress at late time points (Fig. 6A). In shoot, there were some early responsive *CeqWRKY* genes, such as *CeqWRKY17*, *CeqWRKY18*, *CeqWRKY22*, *CeqWRKY25*, *CeqWRKY33*, *CeqWRKY40*, *CeqWRKY41*, *CeqWRKY46*, *CeqWRKY53*, and *CeqWRKY61* (Fig. 6B). Compared to the expression pattern of *CeqWRKYs* in root, no *CeqWRKY* genes displayed significant late response to NaHCO_3_ stress in shoot (Fig. 6B). Additionally, some *CeqWRKYs* maintained continuous induction under NaHCO_3_ stress, such as *CeqWRKY4*, *CeqWRKY25,* and *CeqWRKY62* in root, and *CeqWRKY17*, *CeqWRKY22*, *CeqWRKY28*, *CeqWRKY29*, *CeqWRKY31*, *CeqWRKY39*, *CeqWRKY61*, and *CeqWRKY65* in shoot (Fig. 6).

Since salinization and alkalization often occur simultaneously in natural soil environments [49], it was important to identify a set of genes induced by both NaCl and NaHCO_3_ stresses. To this end, we identified 20 *CeqWRKYs* responsive to both stresses, such as *CeqWRKY11*, *CeqWRKY28*, *CeqWRKY29*, *CeqWRKY31*, *CeqWRKY33*, *CeqWRKY41*, *CeqWRKY45*, *CeqWRKY46*, and *CeqWRKY50*, etc. (Figs. 5 and 6). In addition, some *CeqWRKY* genes displayed different response under NaCl and NaHCO_3_ stresses. For instance, *CeqWRKY21*, *CeqWRKY23*, and *CeqWRKY47* were only involved in response to NaHCO_3_ stress both in root and shoot, but not to NaCl stress (Figs. 5 and 6).

### Co-expression networks analysis of *CeqWRKYs*

**Fig. 7:**
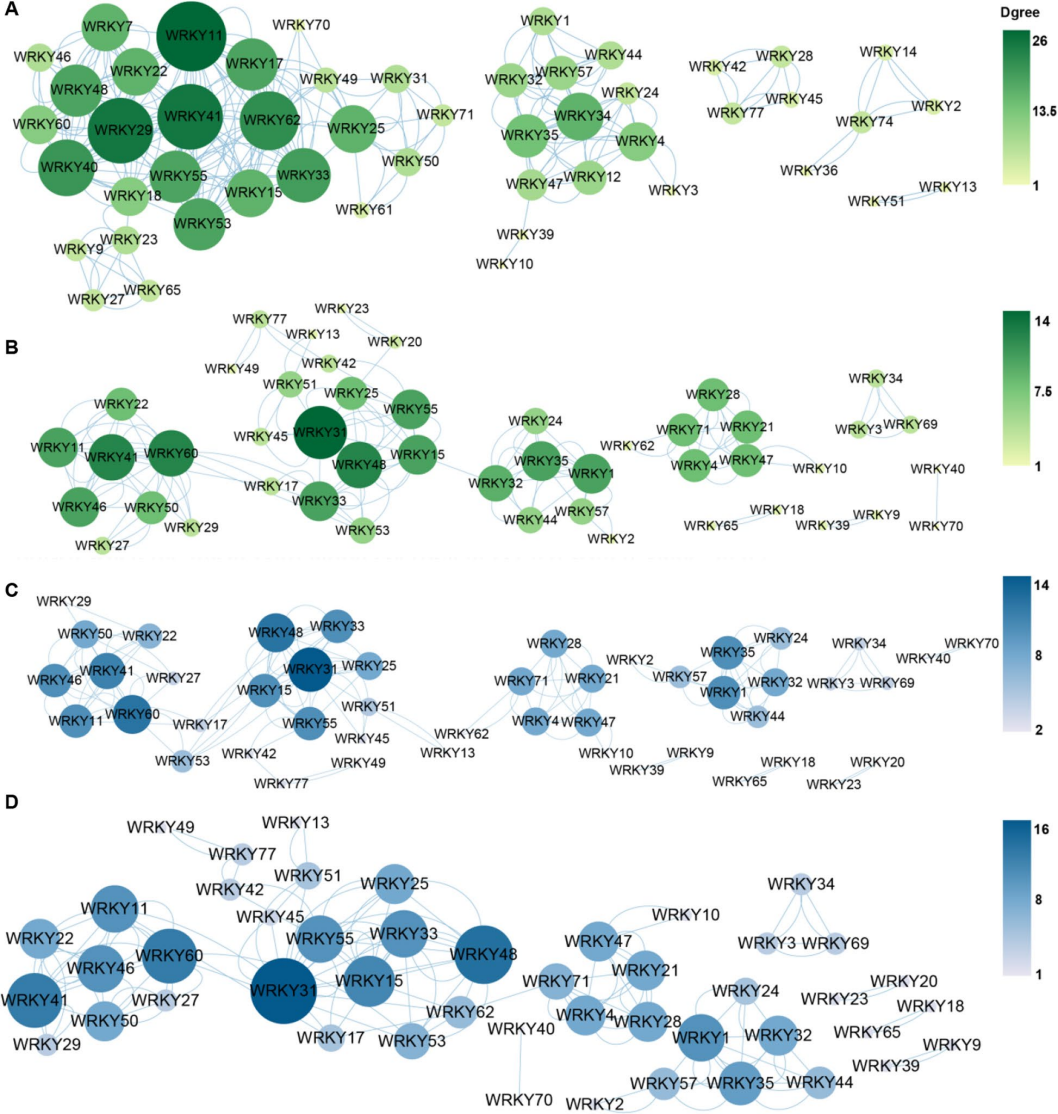
Co-expression networks among *CeqWRKYs* based on the gene expression in transcriptome data. **A**-**B** Co-expression networks among *CeqWRKYs* under NaCl stress in root and shoot, respectively. **C**-**D** Co-expression networks among *CeqWRKYs* under NaHCO_3_ condition in root and shoot, respectively. Degree value represents the number of connecting lines around a node (circle). Larger and darker coloured circles indicate strong correlation with other genes. Edges are drawn when the linear correlation coefficient is > 0.8 with *p*-value < 0.05

WRKY TFs regulate the expression of downstream target genes by binding to W-box located in their promoter region [50]. Due to the presence of different number of W-box elements in the *CeqWRKYs’* promoter (Fig. S1). Co-expression networks analysis was performed for all *CeqWRKYs* using transcription expression data under NaCl and NaHCO_3_ stresses in both root and shoot. The co-expression networks data showed that there were complex regulation networks among *CeqWRKYs* and that one *CeqWRKY* could regulate several other *CeqWRKYs* (Fig. 7).

Under NaCl stress, there were several *WRKYs* with higher degree values at the core in the larger network of root, such as *CeqWRKY41*, *CeqWRKY11*, *CeqWRKY29*, *CeqWRKY62*, *CeqWRKY40* and *CeqWRKY33*. In the smaller network, *CeqWRKY34* may take key parts in response to salt stress in root (Fig. 7A)*.* In shoot, the putative co-expression network showed relatively dispersed, compared to that in root, and the *CeqWRKY* genes located in the center position of each network was different, including *CeqWRKY41, CeqWRKY31,* and *CeqWRKY35* (Fig. 7B)*.* These indicated that *CeqWRKYs* could form different regulatory networks through interaction with other CeqWRKYs under salt stress response.

Under NaHCO_3_ stress, *CeqWRKY41*, *CeqWRKY46*, *CeqWRKY60*, *CeqWRKY11*, *CeqWRKY31*, *CeqWRKY48*, and *CeqWRKY33* may function as the key regulators to mediate NaHCO_3_ stress response in root (Fig. 7C). In shoot, the core genes were same as those in root, but had higher value in shoot than in root. Besides that, *CeqWRKY1* and *CeqWRKY35* also had higher degree value in shoot (Fig. 7D).

### The analysis of subcellular localization and transcriptional activation activity of CeqWRKYs

**Fig. 8:**
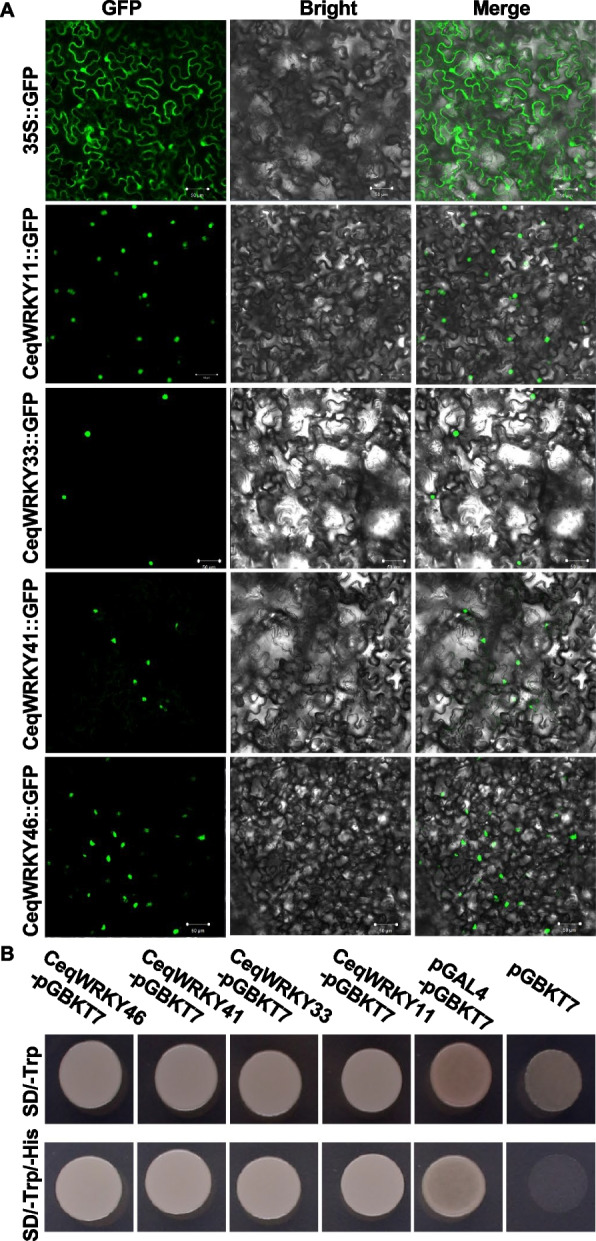
Subcellular localization and transcriptional activation activity analysis. **A** Subcellular localization analysis of CeqWRKY11, CeqWRKY33, CeqWRKY41, and CeqWRKY46. **B** Transcriptional activation activity analysis of CeqWRKY11, CeqWRKY33, CeqWRKY41, and CeqWRKY46

Previous studies showed that WRKY11, WRKY33, WRKY41, and WRKY46 from different plant species play key roles in response to diverse stresses [47–49]. According to the co-expression networks, these four *CeqWRKYs* may play important roles in response to NaCl and NaHCO_3_ stress. To verify whether these WRKYs function as transcription factors in *C. equisetifolia*, the subcellular localization of the above mentioned WRKYs was analyzed. The constructs (35S-CeqWRKYs::GFP) of these genes were separately transformed into tobacco leaves by *Agrobacterium tumefaciens-*mediated transient method. Compared to the empty vector control, the GFP signal from different CeqWRKYs fused proteins was detected only in the nucleus (Fig. 8A), indicating that CeqWRKY11, CeqWRKY33, CeqWRKY41 and CeqWRKY46 were specifically nucleus-localized proteins, and may function as TFs in stress responses.

To further analyze the transcriptional activation activity of these WRKYs, the *pGBKT7-CeqWRKYs* plasmids (for *CeqWRKY11*, *CeqWRKY33*, *CeqWRKY41* and *CeqWRKY46*) were transformed into AH109 yeast cells. pGAL4 and empty vector pGBKT7 were used as positive and negative control, respectively. All the transformants exhibited growth on the SD/-Trp plate (Fig. 8B). Moreover, the transformants carrying the *pGBKT7-CeqWRKYs* plasmids and the positive control grew well on SD/-Trp/-His medium, whereas the negative control did not grow (Fig. 8B). These data demonstrated that the CeqWRKYs could exhibit remarkable self-activation activity.

### Phenotype analysis of transgenic *C. equisetifolia* plants with overexpressing *CeqWRKY11* under NaCl and NaHCO_3_ stresses

**Fig. 9:**
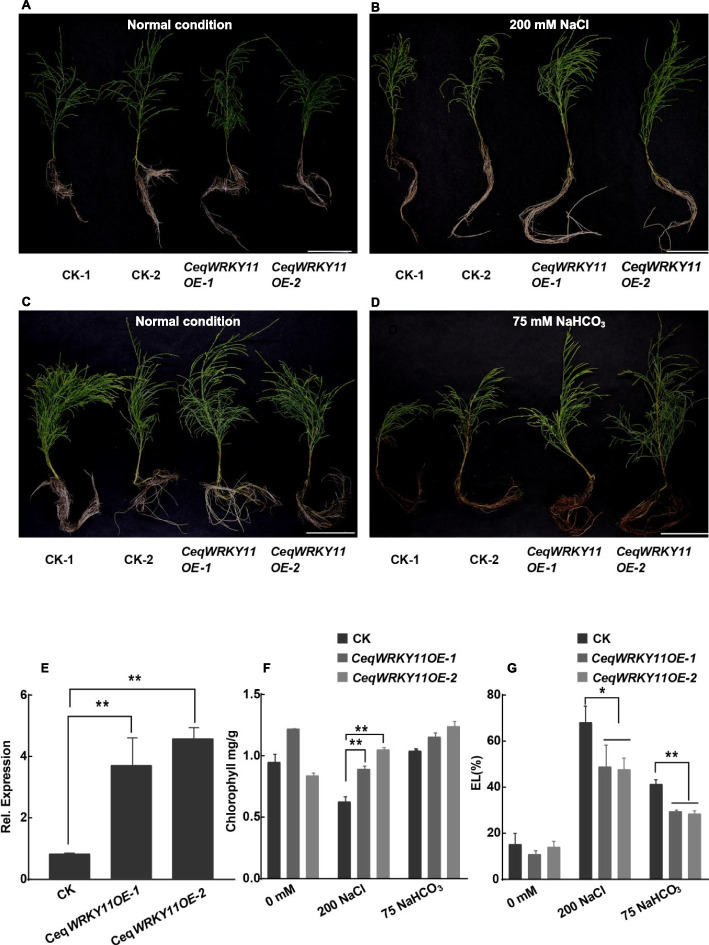
Phenotype analysis of *CeqWRKY11*-overexpressing transgenic plants (*CeqWRKY11OE-1*, *CeqWRKY11OE-2*) in *C.equisetifolia* under NaCl and NaHCO_3_ condition. **A**-**B** Phenotype analysis of *CeqWRKY11*-overexpressing transgenic plants under normal and 200 mM NaCl condition. **C**-**D** Phenotype analysis of *CeqWRKY11*-overexpressing transgenic plants under normal and 75 mM NaHCO_3_ condition. **E** The expression of *CeqWRKY11* in control and *CeqWRKY11*-overexpressing transgenic plants. **F** Chlorophyll content in control and *CeqWRKY11*-overexpressing transgenic plants under normal, 200 mM NaCl and 75 mM NaHCO_3_ condition. **G** Electronic leakage of control and *CeqWRKY11*-overexpressing transgenic plants under normal, 200 mM NaCl and 75 mM NaHCO_3_ condition. T-tests were used to indicate significant difference: * indicates *p* < 0.05, and ** indicates *p* < 0.01

To further study the function of *CeqWRKY11* in NaCl and NaHCO_3_ response, transgenic *C. equisetifolia* plants with overexpressing *CeqWRKY11* were developed by hairy-root mediated transgenic method, and their phenotype was analyzed under 200 mM NaCl and 75 mM NaHCO_3_ conditions, respectively. Considering the fusion of CeqWRKY11 and GFP protein in recombinant plasmid, the GFP fluorescence was detected to identify the transgenic positive *C. equisetifolia* plants. The results showed that, compared to the negative control (WT), there was obvious green fluorescence in overexpressing *CeqWRKY11* lines and the positive control (with empty overexpressing vector) (Fig. S3). The expression of *CeqWRKY11* in transgenic line roots (*WRKY11OE-1, WRKY11OE-2*) was confirmed by qRT-PCR, and was found to be higher than control plants (CK: transformed with empty vector) (Fig. 9E), demonstrating the overexpression of *CeqWRKY11* in transgenic positive lines. Under normal growth condition, no significant difference was observed in the growth of transgenic plants compared to control (Fig. 9A, C). However, after 5 days of NaCl treatment, the shoots of the CK were severely wilted, but the shoots of overexpression lines grew normally and displayed much greener (Fig. 9B). When grown under NaHCO_3_ treatment for 15 days, the roots of the CK plants showed severe browning, but *WRKY11OE* displayed normal growth without significant changes (Fig. 9D). In addition, there was more abundant chlorophyll content in shoots of the overexpression lines than in control plants under NaCl condition, while there was no significant difference in chlorophyll content between control plants and *WRKY11* overexpression plants under NaHCO_3_ treatment (Fig. 9F). After NaCl or NaHCO_3_ treatment, although the electrolyte leakage was obviously increased in both the overexpression lines and control plants, but compared to control plants, the overexpression lines showed much lower electronic leakage (Fig. 9G). Taken together, these results indicated that the expression of *CeqWRKY11* improves the tolerance of *C. equisetifolia* to NaCl and NaHCO_3_ stresses.

### Overexpression of *CeqWRKY11* altered the expression of stress-responsive genes in *C. equisetifolia*

**Fig. 10:**
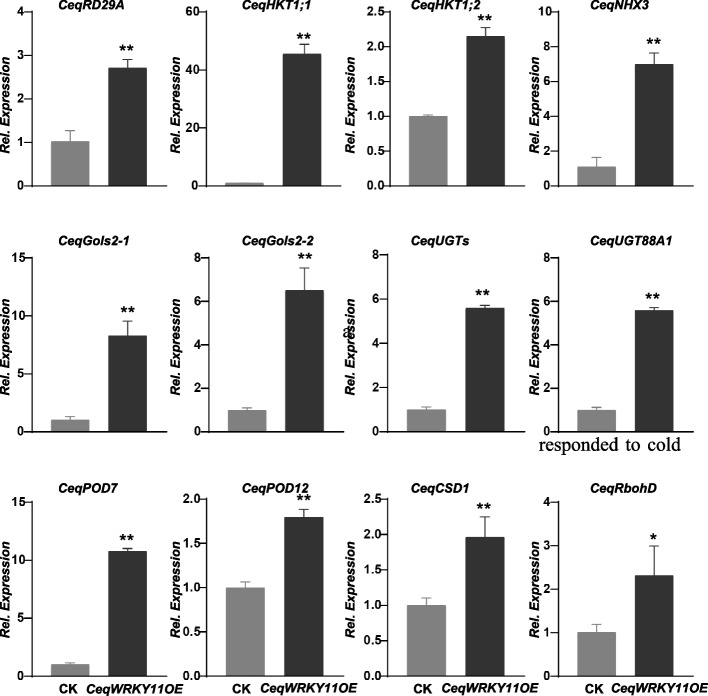
Expression analysis of stress-responsive genes in *CeqWRKY11* overexpression (*OE-WRKY11*) and non-transgenic *C. Equisetifolia* (CK) plants by qRT-PCR. The mean ± standard error measurement (SEM) value with three replications is displayed. Relative expression in CK was set to 1. T-tests were used to indicate significant difference: * indicates *p* < 0.05, and ** indicates *p* < 0.01

To investigate the molecular mechanism of higher NaCl and NaHCO_3_ stress tolerance of *CeqWRKY11* overexpressing lines, we analyzed the expression of several stress-responsive genes (*CeqHKT1*, *CeqNHX, CeqRD29A*, *CeqUGTs*), and oxidative stress related genes (*CeqPOD*, *CeqRbohD*, *CeqCSD1*, *CeqGols*) in the *CeqWRKY11* overexpression lines and control plants under normal condition. The results showed that all these genes, especially *CeqHKT1* and *CeqPOD7*, were markedly up-regulated in transgenic lines compared with control (Fig. 10), indicating that the overexpression of *CeqWRKY11* may improve the NaCl and NaHCO_3_ tolerance in *C. equisetifolia* through regulating the Na^+^/K^+^ balance and maintaining a stable ROS level.

## Discussion

The *WRKY* gene family, one of the largest transcription factor families, plays essential roles in various aspects of plant development and their ability to respond to both biotic and abiotic stresses [5, 51]. Previous studies have been identified *WRKY* genes in different plant species, including 75 *WRKYs* in *A. thaliana* [52], 126 in *O. sativa* [53], 81 in *Solanum lycopersicum* [54], 48 in *Camellia japonica* [55], 197 in *Glycine max* [56], and 98 in *Populus trichocarpa* [57]. In this study, a total of 64 *WRKY* genes were identified in *C. equisetifolia* (Fig. 1), which was smaller, compared to the number of *WRKY* members in other species. The *CeqWRKY* members were categorized into three major groups; group II was further divided into five subgroups (Fig. 2). Furthermore, the distribution of *CeqWRKY* genes within each group or subgroup was similar to that of *A. thaliana* and *P. trichocarpa* [1, 57]. Previous study has demonstrated that genes expression and function were highly related to their regulatory elements in promoter [47]. Our study found that the promoter region of group IIc *CeqWRKYs* included more stress-related *cis*-elements (Fig. 3), and the expression of most of the *CeqWRKYs* was induced by at least one stress treatment (Figs. 5 and 6), indicating that group IIc *CeqWRKYs* could be involved in mediating abiotic stress resistance in *C. equisetifolia.*

Plants respond to many environmental stresses by regulating the complex signal transduction pathways. The *cis*-element analysis showed that, compared to the promoter region of *WRKY* genes from *Taraxacum kok-saghyz* and *Liriodendron chinense, CeqWRKYs* promoter region contained much more hormone response elements, including ABRE, MeJA, and stress related elements, such as W-box (WRKY transcription factor binding site), and TC-rich (salt stress-related elements) (Fig. 3B, Figure S1) [46, 58], inferring that *CeqWRKYs* are involved in ABA- and MeJA-mediated stress responses.

Gene expression is highly correlated with gene function [59]. Previous studies showed that genes specifically expressed in the roots were assumed to be key regulators of root development and may play roles in response to various stresses [60, 61]. Our results showed that many *CeqWRKYs* were highly expressed under normal condition in root or shoot, implying a constitutive expression for *WRKYs* in *C. equisetifolia* (Fig. 4A), which may be in accordance with the strong inherent stress resistance of *C. equisetifolia*. In addition, we found that *CeqWRKYs* quickly and continuously responded to NaCl and NaHCO_3_ stress, but delayed responded to cold stress (Fig. 4B, C, D), which may be one of the reasons for its sensitivity to cold stress. The latest research showed that *WRKY*s are mainly involved in response to salt stress [16]. In our study, we found that most of *CeqWRKYs* genes were separately responsive to NaCl or NaHCO_3_ stress at the early and late treatment time points, and some *CeqWRKYs* displayed continuous response (Figs. 5 and 6). Furthermore, there were different *CeqWRKYs* independently involved in NaCl or NaHCO_3_ stress. For instance, *CeqWRKY47* was only induced by NaHCO_3_ stress, while *CeqWRKY24* was only up-regulated by NaCl stress (Figs. 5 and 6). In addition, the induced expression of *CeqWRKYs* also displayed tissue specificity. Under NaCl stress, *CeqWRKY17* was only up-regulated in shoot while *CeqWRKY10* and *CeqWRKY34* were only induced in root (Fig. 5). These results illustrated that *CeqWRKYs* displayed tissue specificity in expression pattern and functional diversification in response to salt stress. Notably, some *CeqWRKYs* respond to NaCl and NaHCO_3_ stresses at the same time. For example, *CeqWRKY11*, *33*, *41*, *46* were significantly up-regulated by NaCl and NaHCO_3_ stresses in both root and shoot. Moreover, these four *CeqWRKYs* were identified as core genes in co-expression networks, implying that these *CeqWRKYs* may play more important role in NaCl and NaHCO_3_ stress response (Figs. 5, 6 and 7). Furthermore, all four *CeqWRKYs* were localized in nucleus and showed self-transcriptional activation activity (Fig. 8). In addition, comparatively less electrolyte leakage and higher chlorophyll content in the *CeqWRKY11* overexpression lines than control under NaCl and NaHCO_3_ treatment condition, respectively (Fig. 9), showed that *CeqWRKY11* may play a positive role in NaCl and NaHCO_3_ stress response in *C. equisetifolia*, which are similar to the functional characterization of *Arabidopsis WRKY11* [62]. Previous study has reported that, under salt stress, *C. equisetifolia* can impede Na^+^ transfer to the shoot through sequestering Na^+^ in root tissue via Na^+^ and K^+^ transport proteins, and the expression of genes related to oxidative stress and detoxification was up-regulated [63, 64]. In our study, the expression of *CeqHKT1* and *CeqPOD7* in overexpressing *WRKY11* transgenic plants was marked increased (Fig. 10), indicating that *C. equisetifolia* response to NaCl and NaHCO_3_ stresses could partly involve in HKT1*-*mediated Na^+^ and K^+^ transport and POD-mediated ROS scavenging activity.

Transcriptional regulation plays significant role in plants response to multiple stresses [65]. Under diverse stresses, different TFs were activated and then immediately induced the expression of the downstream stress-responsive genes, which triggers a set of responses to minimize stress damage. Recent study showed that OsWRKY63-OsWRKY76-OsDREB1B transcriptional regulatory cascade plays key role in chilling tolerance of rice [66]. Based on the *CeqWRKYs*’ co-expression and interaction networks data (Fig. 7, Fig. S2), we hypothesize that CeqWRKY may interact with each other CeqWRKY(s) or form a transcription cascades to regulate the expression of downstream genes to respond to diverse stresses.

## Conclusions

We identified 64 *WRKY* genes in *C. equisetifolia* and divided into three major groups. Group II was further divided into 5 subgroups. The *CeqWRKY* genes were highly similar with the conserved domains. Their promoter regions contained various response elements. According to the gene expression analysis, *WRKYs* showed delayed transcriptional response to cold stress while a rapid response with varying degrees to NaCl and NaHCO_3_ stresses. *CeqWRKYs* may take important parts in response to NaCl and NaHCO_3_ stresses through the interaction among CeqWRKYs. Furthermore, overexpression of *CeqWRKY11* enhanced the tolerance of transgenic *C. equisetifolia* seedlings to NaCl and NaHCO_3_ stress by up-regulation the expression of abiotic stress related genes, especially *CeqHKT1* and *CeqPOD7*. This systematic analysis of *CeqWRKY* gene family will provide a foundation for the function and regulatory mechanism of the *WRKYs* in abiotic stress response and a theoretical basis for forest trees improvement.

### Supplementary Information

**Supplementary Material 1.**

## Data Availability

All data analyzed during this study are included in this article and its additional files.
