# Supplementary material for: Genome-wide identification of WRKY transcription factors in Casuarina equisetifolia and the function analysis of CeqWRKY11 in response to NaCl/NaHCO3 stresses
Source: BMC Plant Biol. 2024 May 8;24:376. doi: 10.1186/s12870-024-04889-w (PMC11077731; doi:10.1186/s12870-024-04889-w)
Supplement: Supplementary file 1 — Supplementary Material 1. [file 12870_2024_4889_MOESM1_ESM.docx]

**Supplementary Table 1. Physical and chemical analysis of WRKY in *Casuarina equisetifolia***

| Name | Gene ID | Group | Molecular weight | PI(isoelectric point） | Subcellular location | amino acids |
| --- | --- | --- | --- | --- | --- | --- |
| *CeqWRKY1*  *CeqWRKY2*  *CeqWRKY3*  *CeqWRKY4*  *CeqWRKY6*  *CeqWRKY7*  *CeqWRKY9*  *CeqWRKY10*  *CeqWRKY11*  *CeqWRKY12*  *CeqWRKY13*  *CeqWRKY14*  *CeqWRKY15*  *CeqWRKY17*  *CeqWRKY18*  *CeqWRKY20*  *CeqWRKY21*  *CeqWRKY22*  *CeqWRKY23*  *CeqWRKY24*  *CeqWRKY25*  *CeqWRKY27*  *CeqWRKY28*  *CeqWRKY29*  *CeqWRKY31*  *CeqWRKY32*  *CeqWRKY33*  *CeqWRKY34*  *CeqWRKY35*  *CeqWRKY36*  *CeqWRKY38*  *CeqWRKY39*  *CeqWRKY40*  *CeqWRKY41*  *CeqWRKY42*  *CeqWRKY43*  *CeqWRKY44*  *CeqWRKY45*  *CeqWRKY46*  *CeqWRKY47*  *CeqWRKY48*  *CeqWRKY49*  *CeqWRKY50*  *CeqWRKY51*  *CeqWRKY53*  *CeqWRKY55*  *CeqWRKY56*  *CeqWRKY57*  *CeqWRKY60*  *CeqWRKY61*  *CeqWRKY62*  *CeqWRKY63*  *CeqWRKY64*  *CeqWRKY65*  *CeqWRKY66*  *CeqWRKY67*  *CeqWRKY69*  *CeqWRKY70*  *CeqWRKY71*  *CeqWRKY72*  *CeqWRKY74*  *CeqWRKY75*  *CeqWRKY76*  *CeqWRKY77* | CCG025826  CCG012818  CCG010523  CCG006318  CCG025713  CCG018273  CCG025968  CCG002946  CCG026819  CCG009338  CCG013168  CCG006262  CCG020295  CCG029103  CCG021170  CCG019426  CCG000986  CCG029041  CCG024493  CCG029044  CCG011999  CCG015567  CCG012310  CCG008056  CCG008589  CCG015998  CCG003169  CCG008960  CCG024481  CCG007974  CCG029353  CCG005302  CCG004828  CCG006645  CCG023779  CCG014256  CCG020333  CCG003601  CCG025231  CCG023999  CCG014414  CCG026290  CCG002329  CCG003290  CCG001376  CCG020988  CCG029506  CCG028034  CCG021169  CCG011304  CCG024798  CCG014977  CCG029356  CCG007054  CCG017087  CCG029355  CCG003374  CCG020989  CCG022037  CCG000216  CCG000409  CCG023495  CCG021171  CCG019863 | Ⅰ  Ⅰ  Ⅰ  Ⅰ  Ⅱ-b  Ⅱ-d  Ⅱ-b  Ⅰ  Ⅱ-d  Ⅱ-c  Ⅱ-a  Ⅱ-e  Ⅱ-d  Ⅱ-d  Ⅱ-a  Ⅰ  Ⅱ-d  Ⅱ-e  Ⅱ-c  Ⅱ-c  Ⅰ  Ⅱ-e  Ⅱ-c  Ⅱ-e  Ⅱ-b  Ⅰ  Ⅰ  Ⅰ  Ⅱ-e  Ⅱ-b  Ⅲ  Ⅱ-d  Ⅱ-a  Ⅲ  Ⅱ-b  Ⅱ-c  Ⅰ  Ⅱ-c  Ⅲ  Ⅱ-b  Ⅱ-c  Ⅱ-c  Ⅱ-c  Ⅱ-c  Ⅲ  Ⅲ  Ⅱ-c  Ⅱ-c  Ⅱ-a  Ⅱ-b  Ⅲ  Ⅲ  Ⅲ  Ⅱ-e  Ⅲ  Ⅲ  Ⅱ-e  Ⅲ  Ⅱ-c  Ⅱ-d  Ⅱ-d  Ⅱ-c  Ⅱ-a  Ⅱ-c | 52.00832  84.76621  62.0856  55.75802  62.05444  38.72972  54.43357  83.28089  31.71911  20.15416  25.8404  47.14218  36.55329  36.47554  33.82793  62.82969  37.63866  38.40317  33.86599  27.63678  64.73273  72.46982  34.73345  31.21407  62.97507  45.63834  61.42612  14.56403  29.12625  60.72132  33.43437  38.22114  34.01988  40.11234  55.17786  24.35754  52.2909  19.02726  39.64504  57.65458  35.69062  32.50596  18.91356  22.16553  53.03554  39.2807  20.06752  38.88681  31.70967  57.84031  33.77963  22.84266  37.02493  28.7666  17.9955  33.50828  24.51728  34.6275  33.42505  64.03707  37.43599  20.13365  33.47378  22.52264 | 5.26  5.93  7.72  6.42  5.76  9.43  6.04  5  9.8  7.72  9.46  5.33  9.68  9.64  6.25  6.86  9.61  5.06  7.65  9.47  6.42  7.01  8.27  5.72  6.23  9.12  6.3  9.36  4.77  7.94  6.25  9.63  8.57  5.62  6.54  9.35  8.94  9.5  5.54  9.08  5.94  5.03  4.94  6.33  5.84  7.17  9.54  5.73  8.48  8.24  5.44  6.15  6.01  4.85  9.45  5.36  5.78  5.68  6.72  7.15  9.75  9.45  6.83  9.54 | nucleus  nucleus  nucleus  nucleus  nucleus  nucleus  nucleus  nucleus,cytoplasm  nucleus  nucleus  nucleus  nucleus  nucleus  nucleus  nucleus  nucleus  nucleus  nucleus  nucleus  nucleus  nucleus  nucleus  nucleus  nucleus  nucleus  nucleus  nucleus  nucleus  nucleus  nucleus  nucleus  nucleus  nucleus  nucleus  nucleus  nucleus  nucleus  nucleus  nucleus  nucleus  nucleus  nucleus  nucleus  nucleus  nucleus  nucleus  nucleus  nucleus  nucleus  nucleus  nucleus  nucleus  nucleus  nucleus  nucleus  nucleus  nucleus  nucleus,cytoplasm  nucleus  nucleus  nucleus  nucleus  nucleus  nucleus | 474  788  565  520  571  359  503  760  320  184  234  436  332  341  305  581  337  350  305  247  591  660  311  280  585  417  558  131  260  560  299  334  309  358  502  218  480  170  356  540  291  166  196  475  361  176  354  285  538  304  202  330  257  164  299  230  310  301  588  334  178  298  205 |

**Supplementary Table 2. The 20 motifs in *Casuarina equisetifolia* WRKY proteins**

| Motif  Number | Motif Sequence | Motif Logo | Width |
| --- | --- | --- | --- |
| Motif1  Motif2  Motif3  Motif4  Motif5  Motif6  Motif7  Motif8  Motif9  Motif10  Motif11  Motif12  Motif13  Motif14  Motif15  Motif16  Motif17  Motif18  Motif19  Motif20 | YRWRKYGQKPIKGSPYPRSYYRCT  GCPVRKQVZRSSEDPSILITTYEGEHNHP  DGYNWRKYGQKQVKGSEFPRSYYKCTHPNCPVK  KKVERSLDGQITEIIYKKEKKKGZKKVREPRVAVQTRSEVDILDDG  AIEELVQGRECANQLRELLQKSPGDYGSAPAKDLALKILNSFSNTISILN  MVHNQSSSYDYLEAPDQTAFGSLCNMTALPSPMESDDCDDISGFLLDTFN  RCHCSKKRKPRVKRVIKVPAISPKLADIPPDE  VEAATAAJTADPNFTAALAAAISSIIGD  KEELQLLEAELGRVKEENKRLKEMLDQVAKBYNALQMHLVEJLQKNEAK  PFPNAPEQILASSPGDSPFVLSFENSLTSKQNHPFLSSFQPAKEEHKEAI  LPPAATAMASTTSAAASMLLSGSSSS  TLKEPRGCRKSRVTVQTESSDTSLLVDDG  YAWRKYGQKAIKNAKFPRYT  SSMSSISASSPFPTITLDLT  GQHNHPKPQPNRRS  YLTIPPGLSPTTLLESPVLL  LTAITDVAVSKFKKVISLLNR  SKKRKSLQKRVVQVPIADGEG  ENGWGWEQATLIAELTQGMELAKQLRKHLN  IEKSSDNFEHILSQMQIY | 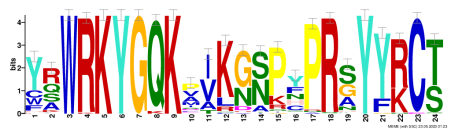  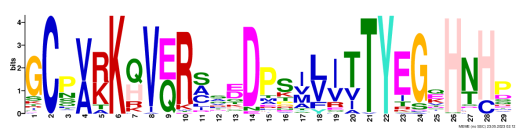  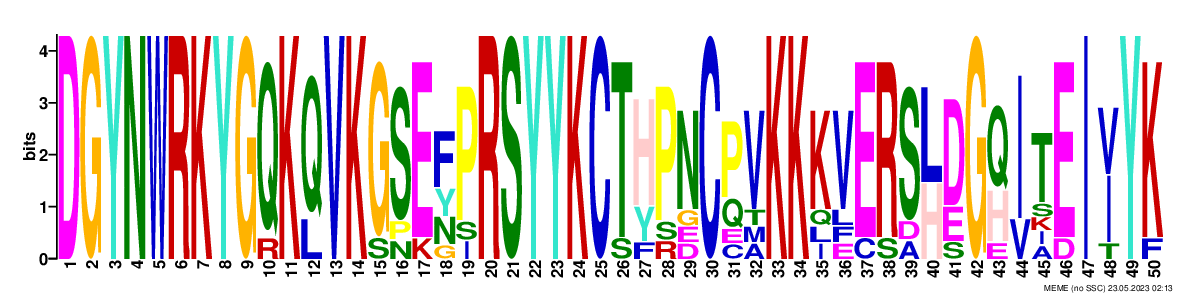  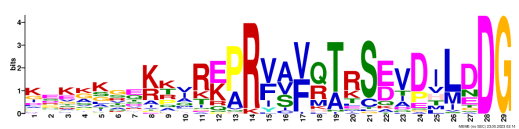  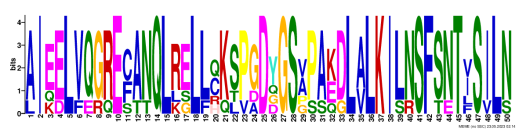  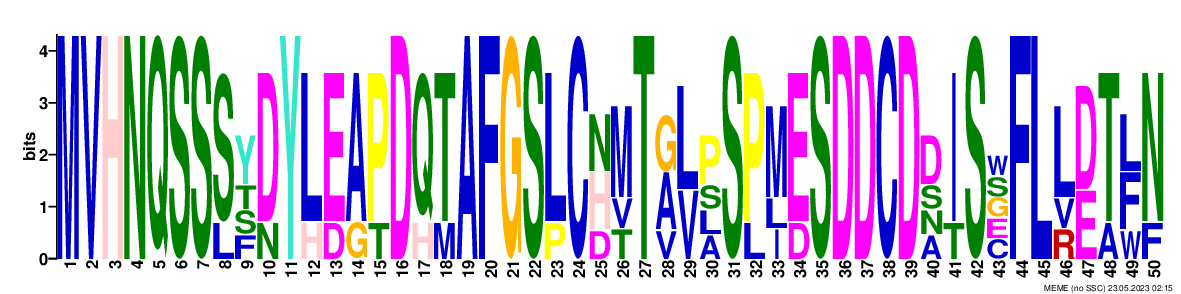  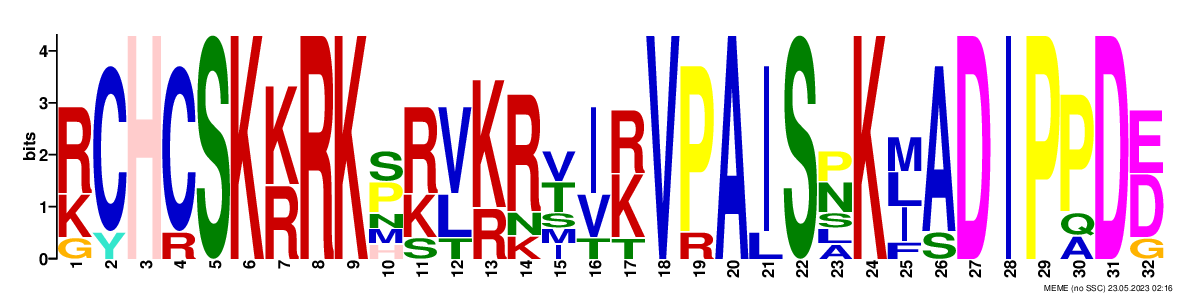  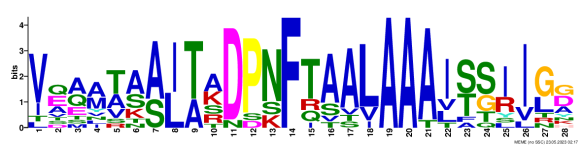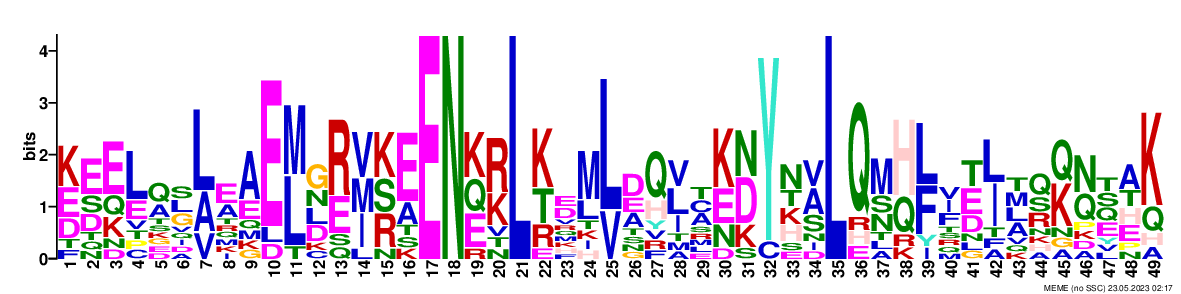  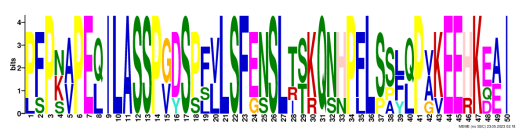  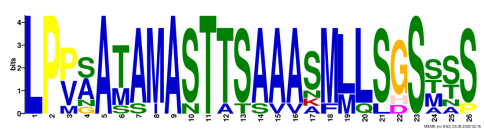  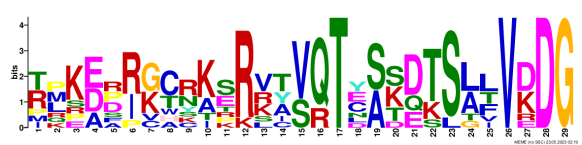  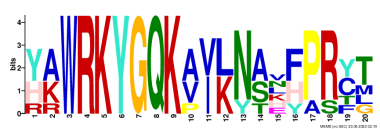  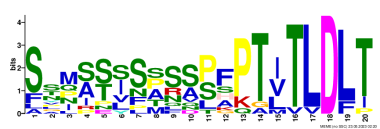  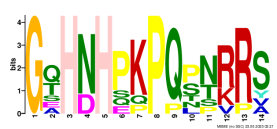  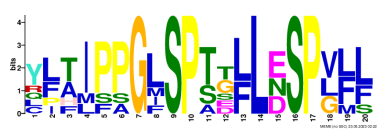  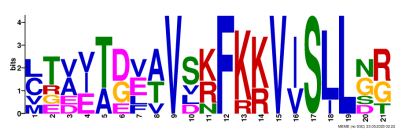  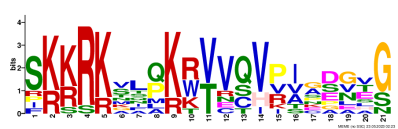  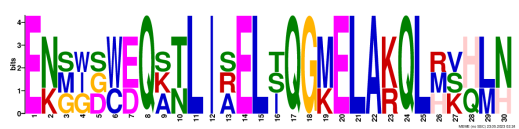  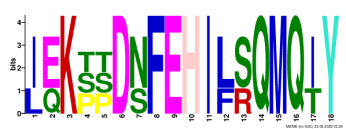 | 24  29  50  29  50  50  32  28  49  50  26  29  20  20  14  20  21  21  30  18 |

**Supplementary Table 3. The expression data of *CeqWRKYs* under NaCl and NaHCO_3_ stresses**

**Supplementary Table 4. The expression data of *CeqWRKYs* under cold stress**

| GENE | CK | 10 min | 2 h | 24 h | 168 h |
| --- | --- | --- | --- | --- | --- |
| *CeqWRKY1* | 19.50759967 | 17.213936 | 18.84715633 | 12.03286733 | 14.828884 |
| *CeqWRKY2* | 22.27398933 | 20.272756 | 19.71417767 | 15.37492533 | 9.377898 |
| *CeqWRKY3* | 23.04120133 | 18.80079267 | 14.44869667 | 20.386448 | 26.75987667 |
| *CeqWRKY4* | 18.09400667 | 17.289538 | 16.50804 | 16.37611667 | 22.890165 |
| *CeqWRKY6* | 2.541312333 | 2.530532667 | 2.506600667 | 14.545605 | 252.84787 |
| *CeqWRKY7* | 14.726108 | 14.605268 | 19.96924067 | 38.009192 | 158.845983 |
| *CeqWRKY9* | 0.007751 | 0.0001 | 0.0001 | 0.0001 | 0.065282333 |
| *CeqWRKY10* | 0.477221333 | 0.6904 | 0.423557 | 0.201293 | 0.265847333 |
| *CeqWRKY11* | 32.65226267 | 24.528919 | 39.876545 | 108.747574 | 111.356753 |
| *CeqWRKY12* | 5.000557667 | 3.459063667 | 3.576463 | 2.449159333 | 0.719715 |
| *CeqWRKY13* | 16.20888967 | 9.936521333 | 9.780681333 | 4.648495667 | 1.611534667 |
| *CeqWRKY14* | 2.333293333 | 2.903988667 | 1.906164333 | 0.471767333 | 0.202322 |
| *CeqWRKY15* | 40.17084367 | 38.35729967 | 49.19782133 | 33.992754 | 284.1731823 |
| *CeqWRKY17* | 21.615238 | 17.385221 | 14.62411867 | 15.145067 | 27.49610967 |
| *CeqWRKY18* | 0.333544 | 0.358128333 | 0.545540333 | 19.55675 | 37.34525667 |
| *CeqWRKY20* | 26.450285 | 25.29671033 | 24.53029033 | 21.04653433 | 67.13634767 |
| *CeqWRKY21* | 21.64994667 | 16.425819 | 16.11601967 | 9.964179 | 14.483342 |
| *CeqWRKY22* | 3.731409 | 3.177440667 | 3.666223 | 11.08291167 | 98.73109433 |
| *CeqWRKY23* | 1.594736667 | 1.003907667 | 0.971706 | 3.140284 | 13.30699033 |
| *CeqWRKY24* | 4.098370667 | 7.753867 | 7.840275 | 3.252521 | 1.366726 |
| *CeqWRKY25* | 5.229373333 | 5.871979667 | 3.921926667 | 90.42557533 | 370.604421 |
| *CeqWRKY27* | 1.089220333 | 0.672478333 | 0.745273 | 0.652361667 | 1.306098 |
| *CeqWRKY28* | 0.238390667 | 0.492949 | 0.543273 | 0.463025333 | 3.306406 |
| *CeqWRKY29* | 0.0001 | 0.0001 | 0.0001 | 0.059044333 | 10.027179 |
| *CeqWRKY31* | 12.65859433 | 13.145797 | 12.99441567 | 7.013036667 | 79.88827267 |
| *CeqWRKY32* | 33.77686767 | 33.795803 | 32.09304167 | 30.1786 | 58.57149133 |
| *CeqWRKY33* | 2.191106667 | 2.131491 | 3.814211333 | 36.14903467 | 38.268065 |
| *CeqWRKY34* | 0.579488333 | 0.733932667 | 0.419111333 | 0.269119667 | 0.132430333 |
| *CeqWRKY35* | 46.99405033 | 42.83398967 | 20.86943633 | 22.00859067 | 8.405710333 |
| *CeqWRKY36* | 0.0001 | 0.0001 | 0.0001 | 0.0001 | 0.0001 |
| *CeqWRKY39* | 0.826694667 | 0.909086667 | 0.569616 | 0.352522 | 0.824167667 |
| *CeqWRKY40* | 0.62947 | 0.503646333 | 0.58473 | 79.849705 | 169.701406 |
| *CeqWRKY41* | 3.845064 | 4.439254 | 6.091314667 | 12.61602933 | 8.068698333 |
| *CeqWRKY42* | 0.117016667 | 0.115401 | 0.088111667 | 0.656763667 | 0.392777667 |
| *CeqWRKY44* | 73.09328467 | 80.23910733 | 108.2987087 | 74.57773467 | 100.923139 |
| *CeqWRKY45* | 0.537221667 | 0.533526333 | 1.00873 | 0.370293 | 4.646383333 |
| *CeqWRKY46* | 5.53393 | 5.052403 | 5.790086667 | 44.38109333 | 12.42588067 |
| *CeqWRKY47* | 0.282792667 | 0.244303333 | 0.214146 | 0.207780667 | 0.30938 |
| *CeqWRKY48* | 7.044762333 | 4.824691 | 5.783867 | 5.45431 | 63.228859 |
| *CeqWRKY49* | 1.020012333 | 1.807066 | 1.258854 | 0.378626333 | 1.954038 |
| *CeqWRKY50* | 0.055662 | 0.080908667 | 0.0001 | 0.252798 | 0.716041667 |
| *CeqWRKY51* | 1.075407 | 0.723091667 | 0.593480667 | 0.916337667 | 0.214097333 |
| *CeqWRKY53* | 0.024486667 | 0.007514667 | 0.041723 | 0.228369667 | 1.193955667 |
| *CeqWRKY55* | 0.07262 | 0.068975333 | 0.0001 | 0.0001 | 0.046966333 |
| *CeqWRKY57* | 6.451319333 | 5.919834333 | 2.749561333 | 3.592222 | 1.054844 |
| *CeqWRKY60* | 0.0001 | 0.0001 | 0.0001 | 0.0001 | 0.036980333 |
| *CeqWRKY61* | 0.440534667 | 0.316107333 | 0.255064333 | 0.395371333 | 1.585031667 |
| *CeqWRKY62* | 7.367126333 | 5.230523667 | 5.584399333 | 22.04709967 | 2.882282667 |
| *CeqWRKY65* | 6.925413667 | 6.690656 | 1.636236333 | 25.84078133 | 27.047805 |
| *CeqWRKY69* | 6.514092667 | 3.826073333 | 3.260895667 | 2.295801333 | 2.787718333 |
| *CeqWRKY70* | 3.584489333 | 1.665857 | 3.141139333 | 34.56492467 | 2.668023667 |
| *CeqWRKY71* | 3.466101 | 3.038153333 | 1.710153 | 11.35874867 | 26.49646367 |
| *CeqWRKY72* | 0.008194 | 0.018801667 | 0.0001 | 0.246149667 | 0.112305667 |
| *CeqWRKY74* | 3.531793333 | 2.192002667 | 1.809057333 | 0.556083667 | 0.017517667 |
| *CeqWRKY75* | 0.0001 | 0.105347 | 0.010321 | 0.0001 | 9.489298 |
| *CeqWRKY77* | 0.083246667 | 0.069863 | 0.035153 | 0.0001 | 0.032372 |

**Supplementary Table 5. Primers of *CeqWRKYs* used for quantitative real-time PCR analysis in *Casuarina equisetifolia***

| GENE | Primer-F | Primer-R |
| --- | --- | --- |
| *CeqWRKY4* | GAACCCGCTGCTTCACATAG | GCCCTCTCAACATGCTTACG |
| *CeqWRKY3* | GAAGGCATGCTTCCAACCAA | CTTCTGTCCATACTTGCGCC |
| *CeqWRKY25* | CGCACCCAACAAGCTTTACT | CACACCTGACCCTGTTCTCT |
| *CeqWRKY44* | TGTCCCAACCACAGCAAATG | ATTTCCGCAATCTGGCCATC |
| *CeqWRKY33* | CAGTCGATGCAGCCTTCTTC | TGCTTCCAGAACCTGCAAAC |
| *CeqWRKY20* | CCGAGCGCTTCTAACAACTC | TGGAGCAAACGGTAGTCACT |
| *CeqWRKY2* | TTGGGGAAAGAATTGCAGCC | GCTCCTACTGTCGCCATTTG |
| *CeqWRKY1* | ACCATGACATGCCTCCTTCA | GGACAGCCATTTCAAAGCCA |
| *CeqWRKY32* | TCAAGTTGAAGGTGGGGTGT | CAGGTAGAGTTGGGCTTGGA |
| *CeqWRKY10* | GCAGGGAAGCATATCACGTG | TTTGCCCATATTTCCGCCAG |
| *CeqWRKY46* | TGAGCTGAGTCAAGGGAAGG | GGCGATTCGAGGGAGTTAGA |
| *CeqWRKY41* | CACGACGATGGCTACAGTTG | GGGGTCCTCATCTGCTCTTT |
| *CeqWRKY23* | GGCTACCACTGTGAAGGAGT | TCTCGGCTCTCTCTGTCTCT |
| *CeqWRKY71* | CAGGGTACAGTTCTCAGGGG | GGAGGAGGAAGAGATCGACG |
| *CeqWRKY53* | TCCAGACGACCCTCAAAGTC | GAAGCTGCTCATCCCACTTG |
| *CeqWRKY11* | AGATAGCCGATATACCGCCG | GATGCGGGTGATGATGTTCC |
| *CeqWRKY48* | GAGGACCAGGACCAAGACAA | CTTTACACCACATCCAGCGG |
| *CeqWRKY75* | GGCAGTCGACCTTTCCTTTC | CATCCCACCTTCATTCGCAG |
| *CeqWRKY49* | TAGCCAGCTTATCTCCACCG | TGCCCGTACTTCCTCCATTT |
| *CeqWRKY56* | TTGGAACTGATGCCGGAGAT | TTTGCCCGTACTTCCTCCAT |
| *CeqWRKY21* | ACCACATCCCTTTTCCCCAA | AGCAAGATGGAGAGGGCTTT |
| *CeqWRKY55* | ACCTGGAGAAAATACGGCCA | GATCTGGGTCATCTCTCGGG |
| *CeqWRKY65* | CAACACAACCACGCTAACCA | GCTGAACCAACCAAACTCGT |
| *CeqWRKY15* | ACCTCGTTCATGTCGTCCTT | TAATCGCGGGGACTCTAACC |
| *CeqWRKY28* | GCTTCACTGACTGCTTGCTT | CTCTTGGCGGATTCTTGCTC |
| *CeqWRKY45* | CGGAGGGTCTGCAGTTTCTA | TGCTCCTTGTTTGAAACGCA |
| *CeqWRKY7* | CTCAAGACCCAGACCCAGAG | TGGTGGAGAAGGCGAGAAAT |
| *CeqWRKY29* | TGGTTCAGATCTGTGGGCAT | TCAGGTGTCCGTAACTTGCT |
| *CeqWRKY24* | CCCTTGTCTCAACCCCAGAT | ACGGAAGGGAGGATGTGAAG |
| *CeqWRKY27* | GGTACGGTGGAGATGGAGAG | GGAATCTGAACCTCGTCCCA |
| *CeqWRKY14* | CTTGATGCATCGGGTTCCAG | GACACCGGCAACTTTGAACT |
| *CeqWRKY74* | TCAAACTCCTGGATGCCGAT | GGGGTTGTGTTGGATTGCAT |
| *CeqWRKY22* | TGTCAAGTACCAGCAGAGGG | GTGGACCCAGCTAGTGAGTT |
| *CeqWRKY57* | CGATGGCGCAAATATGGACA | CTGCCCAGCGAAGTTTTCAT |
| *CeqWRKY50* | GCGAATGAAGAGGATGAGCC | ACTTGAACCCATCGTCCAGT |
| *CeqWRKY17* | GTACCTCAGGCTCCGATCTC | TGATTGCCGACGACATGAAC |
| *CeqWRKY39* | CATCAAAGTGCCTGCCATCA | GATCCTCCACGCATCTCTCA |
| *CeqWRKY35* | TCTCCCAACCCAGACAGAAC | AAGTGAGGGGCTCTTCATCC |
| *CeqWRKY69* | GTGACGTGGAGGGATCTAGG | GGGGAGGGATGGTTATGGTC |
| *CeqWRKY72* | GAGACAATGAAGGCTCGCAG | GCTCGTGGACATGGATTTCC |
| *CeqWRKY51* | GATGCAGCGTGAAGAAGAGG | GCCTGTAAAGTCCATCCCCT |
| *CeqWRKY62* | AAATTCAAGACGATCCGCCG | TGGTAGGCTGACAGAAAGGG |
| *CeqWRKY31* | TCATCGGACGTTGAGGACAA | ACCTTCTGCAGCCTCATTCT |
| *CeqWRKY61* | ACAAAGGATGAAGCTGGGGA | ACTTCCTCCATTGGCATCCA |
| *CeqWRKY6* | ACTGACAGCTACAATGCCCT | CTCGTCGCTTCTTCTTTCCG |
| *CeqWRKY36* | TCAGCTTTTCCTCGTCGTCT | GTATGAAGTGAGTGCAGCGG |
| *CeqWRKY9* | ACGATGAGGCTGGTCAAGAA | GACGTAAAACTCGCCGACTC |
| *CeqWRKY60* | GTGTACGGACCAATGCATCC | TGTGGTTGTGCTCTCCTTCA |
| *CeqWRKY70* | TCCCTGCTCCATAAAACCGT | TTGGAAGCGGACCTCTTTCT |
| *CeqWRKY42* | GAAACACAAGCACGGTTCCT | CGAACATTGAGAGGAAGCGG |
| *CeqWRKY18* | CTCCCACAGTGACCCTTGAT | AGTGCTGCTGTGAAATTGGG |
| *CeqWRKY38* | CCCGTCATCAAACCCGATTC | CGGCGGATCGTCTTGAATTT |
| *CeqWRKY40* | TTCACTTCCTTCCTCGGTCC | AGTGCTGCTGTGAAATTGGG |
| *CeqWRKY47* | CACTAGCAACCAACCACACC | TGGGCTTTTCTTCGTCTTGC |
| *CeqWRKY76* | GCACGGATGTCTGAGTTTCC | CCAAGATAACCATGAGCGGC |
| *CeqWRKY63* | ACATGTCGGCTGTTCCCTAA | TGTGGCAGAGTGATCCGAAT |
| *CeqWRKY43* | AGCTGACGGAGGAGGAGATA | ATTTCTGGCCGTACTTTCGC |
| *CeqWRKY34* | AGCTTCCATGGAGATCGAGG | GAAGATGGAGCCTGCGATTG |
| *CeqWRKY67* | AAATTCAAGACGATCCGCCG | TTGTGTTCCTCTTTTGCCGG |
| *CeqWRKY64* | GCTGGACTGAGAGTTGGGAT | CCTCTCCATTCTGCAGGTGA |
| *CeqWRKY77* | GTTTGCGTTTCAGACTCGGA | AACTTCTTGGCCAGGGATGA |
| *CeqWRKY12* | CAGGTTCTCAGCTTCTTGGC | ATTGACTGCCTTGGGATCCA |
| *CeqWRKY66* | CGCAAAGTCGGACTCTTACG | GGTACCTCCTGCATTGCTTG |
| *CeqWRKY13* | ACGTCCCAAGGTATGCTCAA | CAGTAGGGCTTGGCTGAGAT |
| *CeqEF1α* | TGGCATGTACTTGAGGATCACA | TGCATTGATGGCACGAAGC |

**Supplementary Table 6. Primers of several *CeqWRKYs* full-length CDS in *Casuarina equisetifolia***

| GENE | Primer-F | Primer-R |
| --- | --- | --- |
| *pCAMBIA1300-35S-CeqWRKY11*::*GFP* | TTGGATCCATGGCGATAGATCTGAGCG | TTTCTAGAAGCGGACTGAAGCACGAAAC |
| *pCAMBIA1300-35S-CeqWRKY33*::*GFP* | TTGGATCCATGTCCTTTGTCGAGCTTCT | TTTCTAGAGCAATCCCCGGAGAGCAATG |
| *pCAMBIA1300-35S-CeqWRKY41*::*GFP* | TTGGATCCATGGAGAACGGTTGGAGTTG | TTTCTAGAAGGGAAAAAAGCTGGGGAAT |
| *pCAMBIA1300-35S-CeqWRKY46*::*GFP* | TTGGATCCATGGAAAAGAGTATAGGTTG | TTTCTAGAGGCGAAGAAGTCTGTGTGAT |
| *CeqWRKY11-pGBKT7* | atggccatggaggccgaattcATGGCGATAGATCTGAGCGAGG | ccgctgcaggtcgacggatccTCAAGCGGACTGAAGCACG |
| *CeqWRKY33-pGBKT7* | atggccatggaggccgaattcATGTCCTTTGTCGAGCTTCTTTCC | ccgctgcaggtcgacggatccTTAGCAATCCCCGGAGAGC |
| *CeqWRKY41-pGBKT7* | atggccatggaggccgaattcATGGAGAACGGTTGGAGTTGG | ccgctgcaggtcgacggatccTCAAGGGAAAAAAGCTGGGG |
| *CeqWRKY46-pGBKT7* | atggccatggaggccgaattcATGGAAAAGAGTATAGGTTGCGATC | ccgctgcaggtcgacggatccTTAGGCGAAGAAGTCTGTGTGATC |

**Supplementary Table 7. The quality evaluation of total RNA**

| Sample | O.D. 260/280 | O.D.260/230 | Conc. (ng/µL) |
| --- | --- | --- | --- |
| CK-R | 2.14 | 1.33 | 316.4 |
| CK-S | 2.08 | 1.34 | 580.6 |
| NaCl-0.5-R | 2.14 | 1.75 | 423.1 |
| NaCl-0.5-S | 2.15 | 1.24 | 781.3 |
| NaHCO_3_-0.5-R | 2.01 | 1.41 | 347.3 |
| NaHCO_3_-0.5-S | 2.15 | 1.56 | 796.6 |
| NaCl-6-R | 2.13 | 1.74 | 251.9 |
| NaCl-6-S | 2.17 | 1.61 | 1490 |
| NaHCO_3_-6-R | 2.05 | 1.12 | 343 |
| NaHCO_3_-6-S | 2.17 | 1.25 | 1598 |
| NaCl-12-R | 2.20 | 0.46 | 301.8 |
| NaCl-12-S | 2.15 | 1.75 | 1407 |
| NaHCO_3_-12-R | 2.15 | 1.83 | 364.6 |
| NaHCO_3_-12-S | 2.18 | 1.86 | 1413 |
| NaCl-24-R | 2.16 | 1.25 | 350 |
| NaCl-24-S | 2.12 | 1.61 | 1019 |
| NaHCO_3_-24-R | 1.93 | 0.91 | 308.6 |
| NaHCO_3_-24-S | 2.13 | 0.93 | 1204 |

**Supplementary Figure 1. The analysis of W-box (TTGACT/C) *cis-*elements of *CeqWRKY* genes promoters**


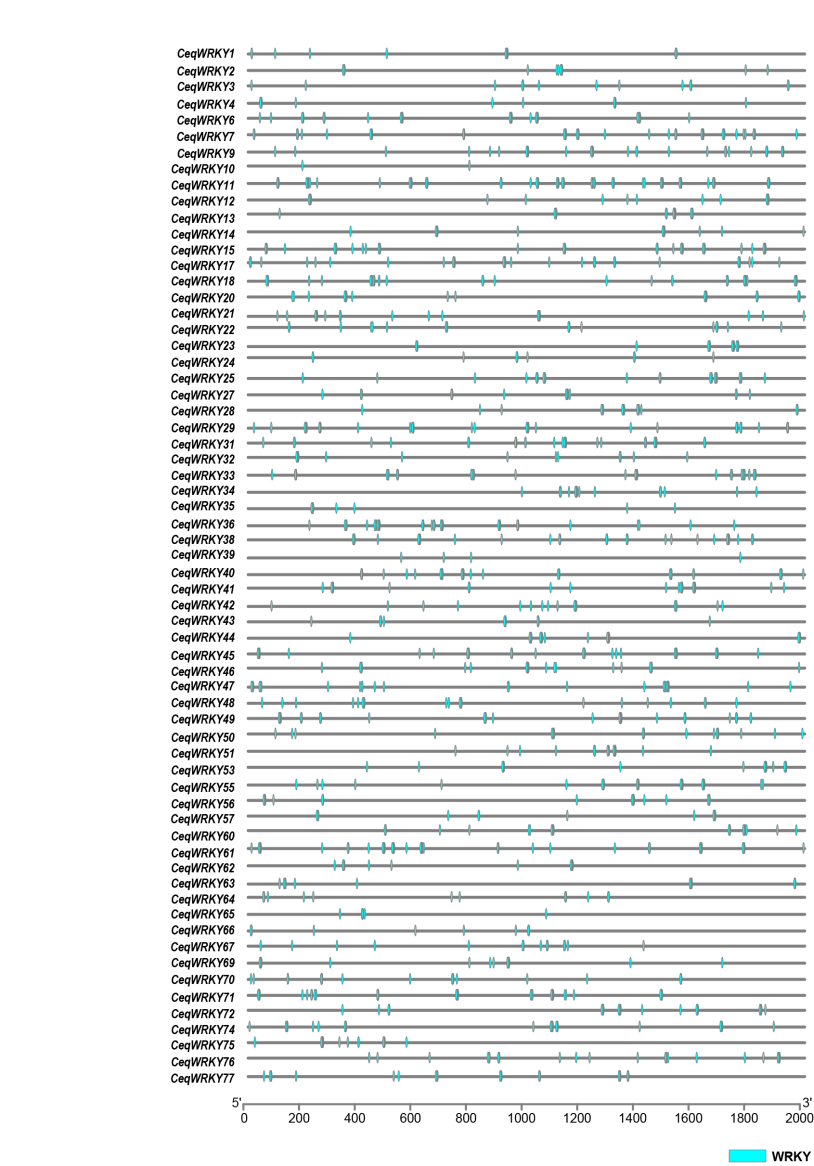


**Supplementary Figure 2. The interaction networks of CeqWRKYs**


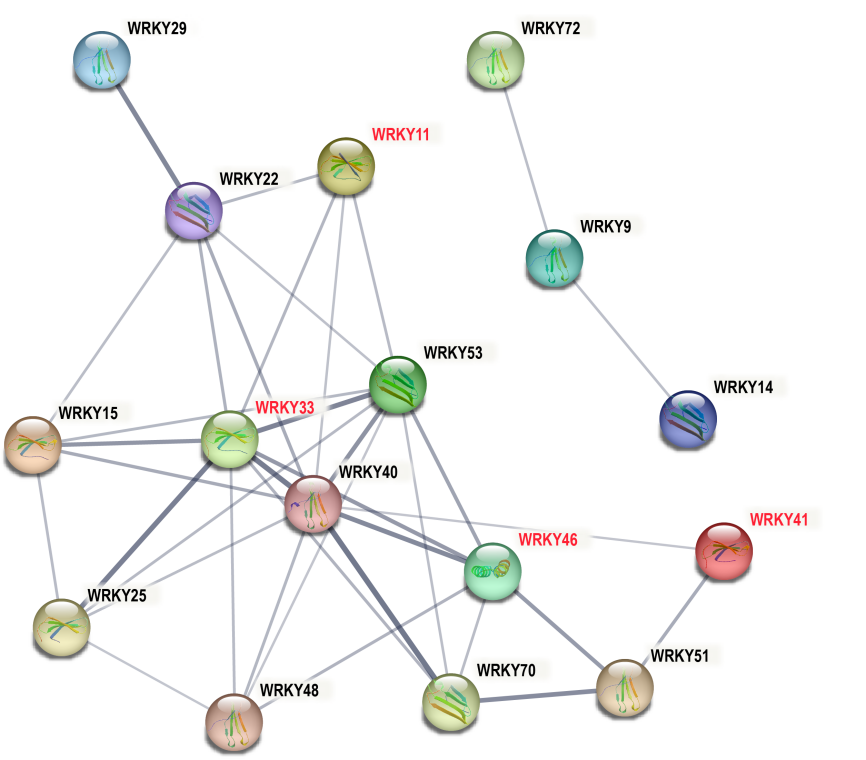


**Supplementary Figure 3. Identification of transgenic *C. equisetifolia* with overexpressing *CeqWRKY11***


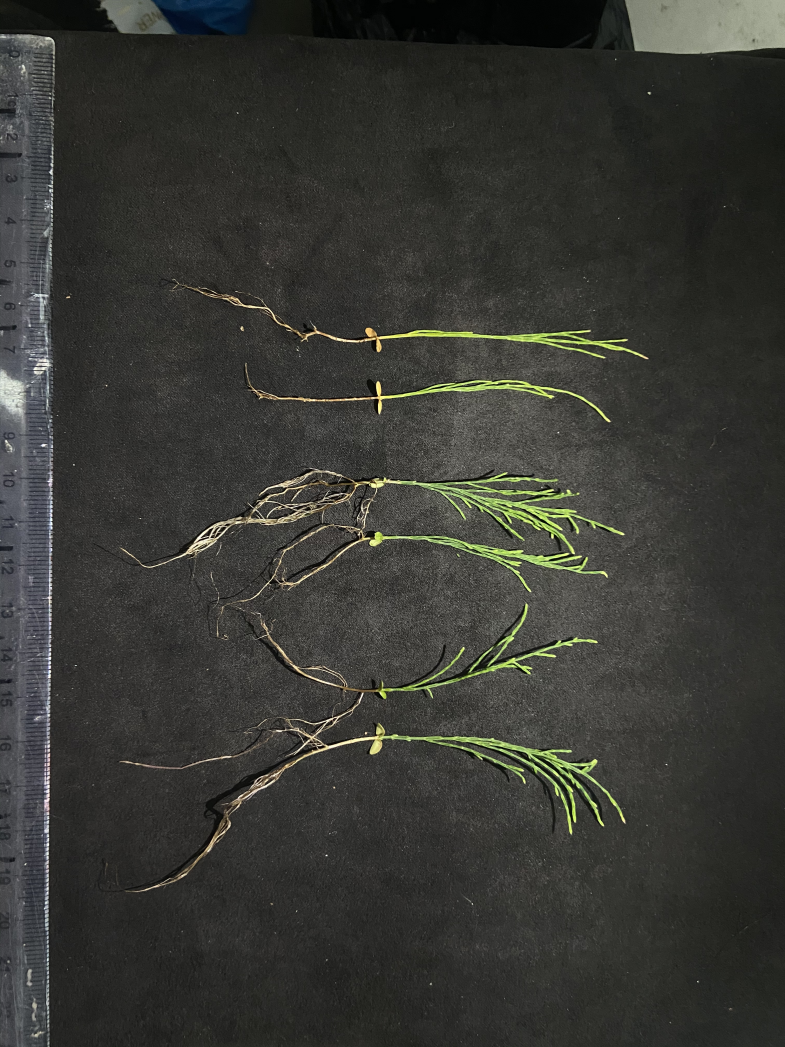

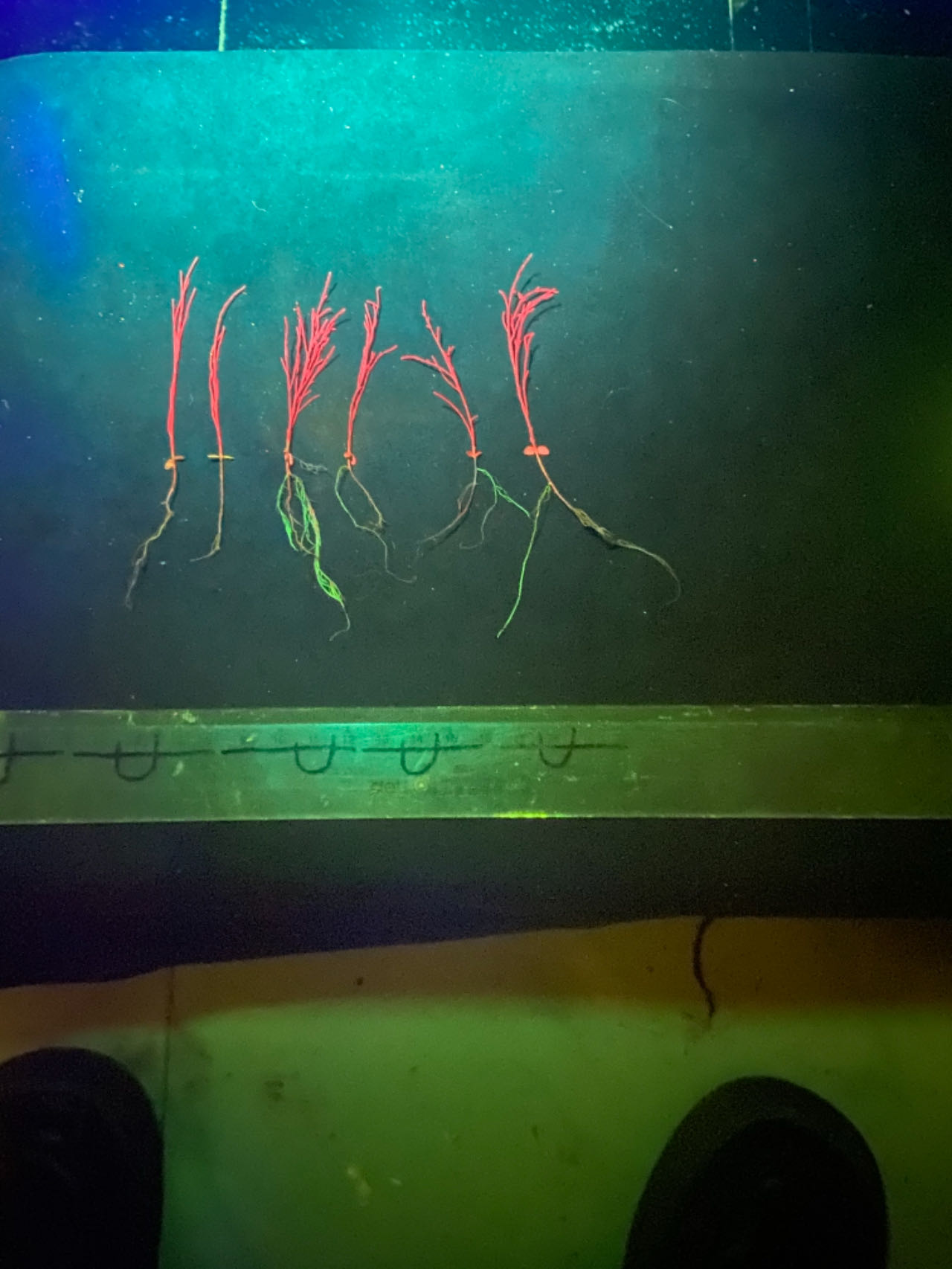


**WT**

**35S::GFP**

**CeqWRKY11::GFP**

**WT**

**35S::GFP**

**CeqWRKY11::GFP**

**1cm**

**1cm**

**Bright**

**Fluorescent**
